# Altered sialidase expression in human myeloid cells undergoing apoptosis and differentiation

**DOI:** 10.1038/s41598-022-18448-6

**Published:** 2022-08-19

**Authors:** Sang W. Hyun, Chiguang Feng, Anguo Liu, Erik P. Lillehoj, Rossana Trotta, Tami J. Kingsbury, Antonino Passaniti, Katerina N. Lugkey, Sitara Chauhan, John F. Cipollo, Irina G. Luzina, Sergei P. Atamas, Alan S. Cross, Simeon E. Goldblum

**Affiliations:** 1 Baltimore Veterans Affairs Medical Center, Research Service, Baltimore, MD 21201 USA; 2 Present Address: Department of Medicine, University of Maryland School of Medicine, HSF2 Room 303, 20 Penn St., Baltimore, MD 21201 USA; 3 Department of Pediatrics, University of Maryland School of Medicine, Baltimore, MD 21201 USA; 4 Department of Pathology, University of Maryland School of Medicine, Baltimore, MD 21201 USA; 5 Department of Physiology, University of Maryland School of Medicine, Baltimore, MD 21201 USA; 6 Center for Vaccine Development and Global Health, University of Maryland School of Medicine, Baltimore, MD 21201 USA; 7 Marlene and Stewart Greenebaum Cancer Center, University of Maryland School of Medicine, Baltimore, MD 21201 USA; 8 Division of Parasitic and Allergenic Products, Center for Biologics Evaluation and Research, Food and Drug Administration, Silver Spring, MD 20993 USA

**Keywords:** Cancer, Immunology, Molecular medicine

## Abstract

To gain insight into sialic acid biology and sialidase/neuraminidase (NEU) expression in mature human neutrophil (PMN)s, we studied NEU activity and expression in PMNs and the HL60 promyelocytic leukemic cell line, and changes that might occur in PMNs undergoing apoptosis and HL60 cells during their differentiation into PMN-like cells. Mature human PMNs contained NEU activity and expressed NEU2, but not NEU1, the NEU1 chaperone, protective protein/cathepsin A(PPCA), NEU3, and NEU4 proteins. In proapoptotic PMNs, NEU2 protein expression increased > 30.0-fold. Granulocyte colony-stimulating factor protected against NEU2 protein upregulation, PMN surface desialylation and apoptosis. In response to 3 distinct differentiating agents, dimethylformamide, dimethylsulfoxide, and retinoic acid, total NEU activity in differentiated HL60 (dHL60) cells was dramatically reduced compared to that of nondifferentiated cells. With differentiation, NEU1 protein levels decreased > 85%, PPCA and NEU2 proteins increased > 12.0-fold, and 3.0-fold, respectively, NEU3 remained unchanged, and NEU4 increased 1.7-fold by day 3, and then returned to baseline. In dHL60 cells, lectin blotting revealed decreased α2,3-linked and increased α2,6-linked sialylation. dHL60 cells displayed increased adhesion to and migration across human bone marrow-derived endothelium and increased bacterial phagocytosis. Therefore, myeloid apoptosis and differentiation provoke changes in NEU catalytic activity and protein expression, surface sialylation, and functional responsiveness.

## Introduction

Polymorphonuclear leukocyte (PMN)s are part of the first line of host defenses against invasive prokaryotic pathogens^4^. Myeloid progenitors undergo maturation within the bone marrow into mature PMNs^1,2^. These motile and highly deformable cells are capable of percolating through and exiting the bone marrow sinusoids, circulating through the intravascular compartment, and recognizing and engaging adhesion molecules surface-expressed on the endothelium. PMNs undergo profound shape changes permitting these cells to squeeze through the small caliber microvasculature and interendothelial cell junctions to enter extravascular tissues^3^, where they adhere to and engulf bacteria for successful phagocytosis and intracellular killing^4^. The HL60 promyelocytic leukemia cell line has been used as a model for myelopoiesis, and its differentiation towards PMN-like cells has been used as a surrogate for short-lived, difficult to manipulate PMNs^5–8^. For example, these same HL60 cells are regularly used in opsonophagocytic assays to establish opsonic activity of vaccine-induced antibodies^9^. Given the critical role of sialic acid (SA), neuraminidase/sialidase (NEU), and sialyltransferase (ST) expression in PMN function^10–13^, we examined their impact on HL60 cellular responsiveness.

SAs comprise a family of 9-carbon sugars, each carboxylated on the C1 position^14,15^. These SA residues are almost always located at the terminus of glycan chains, where they are strategically positioned to influence intermolecular and cell–cell interactions. SA on the cell surface imparts a negative surface charge that generates repulsive forces, influences plasma membrane elastimetry and deformability, motility, adhesion to other cells and substrates, and pseudopod extension and phagocytic function^10,15^. Multiple sialoproteins are expressed on the surface of myeloid cells, and alterations in their sialylation states can explain PMN functional changes. Several examples of such sialoproteins include CD43^16–18^, each of the 2 components of the β2 integrin, CD11b/CD18^11^, CD31^19,20^, and CD44^21–23^. The sialylation state of a specific molecule is dynamically and coordinately regulated through the opposing catalytic activities of STs^24,25^ and NEUs^26–28^. STs catalyze the transfer of SA residues to glycan chains^24,25^, while NEUs hydrolyse the linkage between terminal SAs and their adjacent subterminal sugars^26–28^.

STs are known to influence both in vitro and in vivo PMN behavior^12,29,30^. We previously established PMN surface ST activity with transfer of cytidine monophosphate (CMP)-5-fluorescein isothiocyanate (FITC)-neuraminic acid to the PMN cell surface ^12^. Prior ST inhibition with CMP dramatically reduced transendothelial migration of PMNs, in vitro, and IL-8-induced peritoneal recruitment of PMNs, in vivo^12^. In ST6GAL-1 deficient mice, bone marrow myeloid proliferation and intraperitoneal and circulating levels of PMNs in response to intraperitoneally injected thioglycolate were all increased while apoptosis in elicited PMNs was decreased compared to that observed in wild-type mice^30^. In ST3GAL-4 null mice, PMNs exhibited reduced binding to TNFα-stimulated endothelium, in vitro, and impaired extravasation into the peritoneal cavity, in vivo^29^.

Four human NEUs have been identified, NEU1, 2, 3, and 4^26–28^. Over 25 years ago, prior to the cloning and identification of these four enzymes, we described endogenous PMN NEU activity^31^. We found that upon PMN activation, a preformed pool of NEU activity within one or more intracellular granule subpopulations was rapidly translocated to the plasma membrane, accompanied by SA release. Introduction of exogenous bacterial NEU to PMNs reportedly increases their infiltration of tissues^32,33^, O_2_ and H_2_O_2_ generation^34^, interleukin (IL)-8 release^35^, and extracellular trap formation^35^. Exogenous viral NEU desialylates the PMN surface, provokes their degranulation, and primes these cells for stimulation by galectin 3 and n-formyl-methionyl-leucyl-phenylalanine (fMLP)^36^. We found that exogenous NEU increases PMN adhesion to the endothelium and transendothelial migration of PMNs in vitro^10^, and potentiates LPS-provoked acute lung injury, in vivo^37^. In contrast, prior NEU inhibition diminishes adhesion of activated PMNs to resting endothelial monolayers, in vitro^10^, and PMN recruitment to the lung in response to either cobra venom factor-induced systemic complement activation, or intratracheal installation of IL-8, in vivo^13^. In the SM/J mouse, containing a single amino acid substitution (L209I) in the NEU1 protein, NEU1 activity is markedly reduced^38^. Interestingly, these mice display lower levels of circulating PMNs and reduced PMN interactions with adhesion molecules expressed on tumor necrosis factor (TNF)α-activated endothelium^39^. These combined data indicate that PMNs are equipped with the machinery to both add and remove SA to and from sialylated surface molecules, in turn, influencing PMN function.

In previous studies, differentiation of the HL60 leukemic cell line towards PMNs was associated with altered NEU^5^ and ST^6^ activities. In the current studies, we have established the pattern of NEU and selected ST expression in both resting and proapoptotic mature human PMNs and in the HL60 leukemic cell line during its differentiation towards PMN-like cells. Further, changes in the expression of the chaperone/transport protein for NEU1, protective protein/cathepsin A (PPCA)^40,41^, have been defined. During the differentiation process, changes in sialylation were detected. Finally, we have explored the impact of differentiation on myeloid cell function.

## Materials and methods

### Ethics statement

This study was conducted in accordance with the Declaration of Helsinki and other local statutes or regulations protecting human subjects in biomedical research, and was approved by the Institutional Review Board of the University of Maryland, Baltimore (protocol number HP-00042957). Informed consent was obtained from all participants in this study.

### Human PMN preparation

Whole peripheral blood from healthy human volunteers was collected into acid citrate dextran (Sigma) solution, and PMNs were isolated by dextran erythrocyte sedimentation and density gradient centrifugation through ficoll-hypaque (Sigma), as previously described^10^. PMNs were resuspended in Hank’s balanced salt solution without divalent cations (HBBS) at 10^7^ PMNs/ml and were washed three times with HBSS. For qRT-PCR and immunoblotting experiments, human blood was collected into EDTA-containing vacutainer tubes and PMNs were isolated by negative selection using the Easy Sep Direct Human Neutrophil Isolation kit (Stemcell Technologies, Cambridge, MA). For both isolation procedures, PMN purity was > 95%, and viability > 98% by trypan blue exclusion.

### HL60, human bone marrow microvascular endothelial cell (HBME), and A549 cell cultures and HL60 cell differentiation

HL60 cells were purchased from ATCC (Manassas, VA; certificate of analysis CCL-240, batch #64,048,671), and were maintained in RPMI 1640 medium containing GlutaMax supplement, 10% fetal bovine serum (FBS), and 50 units/ml penicillin and 50 µg/ml streptomycin (Gibco Life Technol Corp). For differentiation, HL60 cells were incubated in the same medium with 0.8% dimethylformamide (DMF) (Sigma), 1.3% dimethyl sulfoxide (DMSO) (Sigma), or 2 µM all-trans retinoic acid (RA) (Sigma) without antibiotics or medium replenishment^9^. A549 cells, an alveolar type II cell derived from a lung adenocarcinoma^42,43^, and human bone marrow microvascular endothelial cell (HBME)s^44^ were maintained in Dulbecco’s modified Eagle’s medium (DMEM) containing 10% FBS, 50 units/ml penicillin, and 50 µg/ml streptomycin, as described^42–45^. HBMEs were used between passages 14–24.

### Fluorometric assay for NEU activity

Human PMNs, HL60 cells, and dHL60 cells were suspended in 200 µl of 500 mM sodium acetate, pH 4.4 containing 0.1% Triton X-100, and a protease inhibitor mixture (Roche Applied Science), and incubated for 1 h at 37 °C with 25 µl of 2.0 mM 2’-(4-methylumbelliferyl)-α-D-*N*-acetylneuraminic acid (4-MU-NANA), mixing the tubes every 15 min, as described^43,45,46^. The NEU reaction was terminated by addition of 133 mM glycine, pH 10.3, 60 mM NaCl, and 0.083 M Na2CO3 after which the fluorescence intensity was measured with a Bio-Rad fluorometer (excitation at 355 nm; emission at 460 nm). In selected experiments, mean NEU activity was normalized to mean cell viability as indicated in the figure legends. In other experiments, cell preparations were preincubated with the broad-spectrum, competitive NEU inhibitor, 2-deoxy-NANA (2DN) (5.0–500 µg/ml)^42,46^. A molecule with comparable molecular weight and charge to 2DN, 2-keto-3-deoxyoctulosonic acid (KDO), was used as a negative control^42,46^.

### qRT-PCR for NEU1-4, PPCA, ST6GAL-1 and ST6GAL-2

Total cellular RNA was extracted from human PMNs, and both nondifferentiated and differentiated HL60 (dHL60) cells, as described^42,43,46,47^. RNA purity was established with the A260/A280 absorption ratio (> 1.90). Total RNA (1.0 µg) was treated with DNase I (Invitrogen) for 15 min and reverse transcribed using avian myeloblastosis virus reverse transcriptase and poly(T) primer (Promega). The resulting cDNA was quantified by real time polymerase chain reaction (qRT-PCR) using SYBR Green PCR Master Mix and an ABI Prism 7900HT cycler. Primers for human NEU1-4, PPCA, ST6GAL-1, and ST6GAL-2 mRNAs were designed using Primer Express 2.0 (Applied Biosystems, Foster City, CA). Relative NEU1-4, PPCA, ST6GAL-1, and ST6GAL-2 gene expression was calculated using the 2^−ΔΔCt^ method where NEU, PPCA, and ST transcripts were normalized to the levels of 18S rRNA transcripts as the internal control, as described^43,47^. Similar amplification rates for all qRT-PCR targets were confirmed by their parallel amplification curves (Fig. S11).

### Immunoblotting for NEU1-4, PPCA, ST6GAL-1, and ST6GAL-2

To prepare PMN lysates, the cells were directly lysed with sodium dodecyl sulfate (SDS)-containing sample buffer in the presence of a protease inhibitor cocktail (Roche Diagnostics, Mannheim, Germany). HL60 and dHL60 cells were thoroughly rinsed with ice-cold HEPES buffer and lysed with ice-cold 50 mM Tris–HCl, pH 8.0, 1.0% Nonidet P-40, 0.5% SDS, 150 mM NaCl, 0.1 mM phenylmethysulfonyl fluoride, 5.0 µg/ml leupeptin, 1.0 mg/ml pepstatin A, 1.0 mg/ml aprotinin, 1.0 mM vanadate, 1.0 mM sodium fluoride, 10 mM disodium pyrophosphate, 500 µM *p*-nitrophenol, and 1.0 mM phenylarsine oxide, as described^19,42,43,45–47^. In selected experiments, PMNs were preincubated with human recombinant granulocyte colony-stimulating factor (G-CSF) (10 ng/ml) (Sigma-Aldrich, St., St Louis, MO). The cell lysates were assayed for protein concentration with the Bio-Rad Protein Assay Dye Reagent. Equal amounts of total cellular protein at 50 µg/lane were resolved by electrophoresis on 8–16% SDS–polyacrylamide gels and transferred to polyvinylidene difluoride (PVDF) membranes. In some experiments, the blots were blocked for 1 h using 5.0% nonfat milk in 50 mM Tris–HCl, pH 8.0, 150 mM NaCl, and 0.01% Tween 20 (TBS-T), probed with murine anti-human NEU1 monoclonal antibody (OriGene, Rockville, MD)^45,47^, rabbit anti-human PPCA monoclonal antibody (Abcam, Cambridge, MA)^45^, rabbit anti-human NEU2 polyclonal antibody (Invitrogen, Thermo Fisher Scientific, Rockford, IL)^48^, rabbit anti-human NEU3 polyclonal antibody (Novus, Centennial, CO)^43^, or rabbit anti-human NEU4 polyclonal antibody (Invitrogen)^48^, each followed by either HRP-conjugated goat anti-rabbit antibody or horse anti-mouse antibody (Cell Signaling, Danvers, MA), and developed with enhanced chemiluminescence (ECL) reagents. Of note, the anti-PPCA antibody recognizes the 54 kDa precursor, not its cleavage products (Abcam product datasheet for ab181129). To confirm equivalent protein loading and transfer, blots were stripped with 62.5 mM Tris–HCl, pH 6.7, 100 mM 2-mercaptoethanol, and 2.0% SDS, washed with TBS-T, reprobed with mouse anti-β-actin antibody followed by horseradish peroxidase (HRP)-conjugated goat anti-mouse antibody and developed with ECL reagents. Immunoblots were captured for quantitative densitometry using a Fujifilm LAS-3000 imaging system and Image Reader LAS-4000 (version 2.1) software (Fuji Medical Systems, Stamford, CT).

### Assays for PMN desialylation and apoptosis

Apoptotic PMNs were detected with the fluorescein isothiocyanate (FITC) Annexin V Apoptosis Detection Kit (Biolegend) in the presence of 7-aminoactinomycin D (7-AAD) to detect nonviable cells, according to the manufacturers’ recommendations. Briefly, purified human PMNs cultured in RPMI 1640 with 10% FBS for increasing times, in the presence or absence of G-CSF (10 ng/ml), were washed twice with cold HBSS^-^, and resuspended in Annexin V Binding Buffer (5 × 10^6^ cells/ml). The PMN suspension (100 µl) was mixed with FITC-annexin V (5 µl) and 7-AAD Viability Staining solution (5 µl) and incubated at room temperature in the dark for 15 min. The stained cells were washed and fixed with 2% paraformaldehyde (PFA) in PBS for 30 min, again washed, and subjected to LSR II flow cytometry (Bectin Dickinson Biosciences). The resultant data were analyzed with Flowjo (Becton Dickinson). The live cell gate was determined by forward scatter (FSC) versus side scatter (SSC) profile. Dead cells were defined as 7-AAD^+^, live cells as 7-AAD^−^ Annexin V^-^, and apoptotic cells as 7-AAD^-^ Annexin V^+^. For peanut agglutinin (PNA) lectin binding as a measure of desialylation, cells were incubated with 5 µg/ml of Alexa Fluor-conjugated PNA (Invitrogen), FITC-annexin V, and 7-AAD, as described above, followed by PFA fixation.

### MTT viability assay

HL60 cells were cultured for 1, 3, 5, and 7 days in the presence of DMF, DMSO, RA, or medium alone. At each time point, the cells were centrifuged (1,500 g, 4 °C, 5 min) and 100 µl of serum-free, phenol red-free RPMI 1640 medium and 10 µl of MTT [3-(4, 5- dimethylthiazol-2-yl)-2,5-diphenyltetrazolium bromide] (5 mg/ml) were added to each cell pellet^45^. As a simultaneous background control, 10 µl of MTT in medium alone without cells, was performed with each assay. The cells were incubated at 37 °C for 3 h, after which 100 µl of DMSO was added, and after 10 min, A_490_ recorded.

### Lectin blotting for sialylated and desialylated molecules

Lysates of HL60 and dHL60 cells, at 50 µg total cellular protein/lane, were resolved by SDS-PAGE and transferred to PVDF membrane. The blots were incubated for 1 h in TBS-T and probed with biotinylated *Maakia amurensis* lectin II (MAL), *Sambucus nigra* agglutinin (SNA), or *Arachis hypogaea* or peanut agglutinin (PNA), as described^19,42,45,46^. The blots were washed with TBS-T, incubated with horseradish peroxidase (HRP)-conjugated streptavidin, and developed with ECL reagents. Fetuin and asialofetuin (1.0 µg each) were used as positive and negative controls for MAL-binding, SNA-binding, and PNA-binding proteins. To confirm equivalent protein loading and transfer, blots were stripped and reprobed for β-actin.

### Selective labeling of oligosaccharide α2,3- and α2,6-linked sialic acid

Selective labeling and stabilization of SAs was performed as previously described^49^. The method consists of glycoprotein immobilization, esterification of α2,6-linked SA, amidation of α2,3-linked SA, and enzymatic release of N-glycans. Briefly, glycoproteins are conjugated to the functionalized aldehydes on the resin via reductive amination. The immobilized glycoproteins are derivatized via esterification using hydroxybenzotriazole hydrate (HBot)/ethanol/N-(3-Dimethylaminopropyl)-N’-ethylcarbodiimide hydrochloride (EDC) resulting in the formation of the ethyl ester on α2,6- linked SAs. Glycoproteins are further modified with p-Toluidine (pT) in the presence of EDC at pH 4–6, resulting in stabilization of α2,3-linked SAs; N-glycans are then released from the solid support (resin) and analyzed by MS.

### MALDI ToF MS analysis of selectively labeled linkage specific sialyl N-glycans

Glycoproteins on the solid support were digested with PNGase F (New England BioLabs Inc., Ipswich, MA). The digestion solution consisted of 1000-unit (1 µL) PNGase F in 300 µL of 20 mM NH4HCO3. The reaction proceeded at 37 °C overnight. Supernatant was collected, and 150 µL of 25% acetonitrile (ACN) was used to wash samples (twice). All washes were pooled with supernatant (a total of 600 µL solution was obtained). Use of the volatile buffer NH4HCO3 allows MALDI-MS analysis of released N-glycans without additional purification. A 4 µL of sample solution was spotted on µ-Focus MALDI plate (Hudson Surface Tech, Fort Lee, NJ), together with 1 µL of DHB (dihydroxybenzoic acid) matrix (10 mg/mL DHB with 2% *N*,*N*’-dimethylaniline (DMA) in 50% ACN and 0.1 mM NaCl). MALDI-MS was performed with a Bruker Autoflex MALDI-TOF MS, each analysis recorded 8000 shots with laser energy at ~ 70%, as described^49^.

### HL60 and dHL60 cell adhesion to HBME monolayers

HBMEs were seeded into 24-well culture plates at 10^5^ cells/well in 1 ml DMEM and cultured to postconfluence (~ 5 × 10^5^ cells/wells). The HBME monolayers were pretreated for 4 h with *Escherichia coli* 0111:B4 lipopolysaccharide (LPS) (Sigma) (200 ng/ml) or medium alone and gently washed. HL60 and dHL60 cells were incubated with 5 µM calcein AM (Molecular Probes, Eugene, OR) for 40 min with gentle agitation in the dark^10^, pretreated for 2 h with 3 nM IL-8 or medium alone, washed, and co-incubated for 0.5 h with HBME monolayers. After gentle washing to remove nonadherent cells, the attached cells were fluorometrically assayed (excitation 485 nm, emission 530 nm) in a Fluoroskan Fluorescence Plate Reader (Thermo Scientific, Ascent, FL), as described^10^.

### Transendothelial migration (TEM) of HL60 and dHL60 cells

TEM of HL60 and dHL60 cells was assayed as previously described^10,12^, with minor modifications. Briefly, sterile collagen-coated Transwell membrane inserts (6.5 mm diameter, 3 µm pore size; Corning, Inc., Kennebunk, ME) which serve as the upper compartment for each assay chamber, were inserted into the wells of 24- well plates, each well serving as the lower compartment for each assay chamber and containing 1.5 mL of DMEM. Each Transwell upper compartment was seeded with 2.0 × 10^5^ HBMEs/ chamber in 0.5 mL of DMEM and cultured to postconfluence (48 h, 37 °C, 5% CO2). These same chambers were inserted into wells containing IL-8 (3 nM) or medium alone. After 2 h, calcein-AM-labeled HL60 or dHL60 cells (5 × 10^5^ cells/well) were introduced into the upper compartments of assay chambers, incubated for 2 h at 37 °C, and the contents of each lower compartment were fluorometrically assayed. The fluorescence of 5 × 10^5^ calcein-AM labeled HL60 or dHL60 cells was used to generate total fluorescence signal in the upper compartment. TEM for each assay chamber was calculated as the ratio of fluorescence intensity in the lower chamber to that measured in the upper chamber × 100%.

### Flow cytometry for expression of CD11c, CD32, gp91^phox^, and for bacterial uptake by nondifferentiated HL60 and dHL60 cells

Nondifferentiated HL60 cells and DMF-differentiated HL60 cells were stained with fluoroprobe-labeled antibodies raised against human CD11c (phycoerythrin-conjugated murine monoclonal anti-human CD11c, Biolegend, San Diego, CA), CD32 (FITC-conjugated murine monoclonal anti-human CD32, Biolegend), and gp91^phox^ (PE-conjugated murine monoclonal anti-human gp91^phox^, LS Bio, Seattle, WA), as well as 7-AAD to exclude dead cells^50,51^. In other experiments, nondifferentiated and DMF-differentiated HL60 cells were incubated in RPMI 1640 + GlutaMAX-1 (Gibco), supplemented with 10% FBS. At the end of the 7-day incubation, cells were harvested, washed with HBSS, counted, and resuspended in opsonophagocytosis buffer (HBSS with Ca^++^Mg^++^, 10% FBS, 0.1% gelatin) to a concentration of 1 × 10^6^/ml. The *Pseudomonas aeruginosa* PAK strain (IATS06), expressing green fluorescent protein (GFP), was streaked onto Trypticase Soy agar containing 100 µg/ml of ampicillin, and incubated overnight at 37 °C. Single colonies were inoculated into fresh Trypticase Soy Broth with ampicillin to reach an OD650 of 0.3 (i.e. bacterial concentration ~ 1 × 10^8^ CFU/ml). The bacteria were washed with HBSS and mixed with the HL60 cell suspension at a multiplicity of infection of 25:1. The HL60 cell/bacteria mixture was incubated at 37 °C for 10–45 min, washed with HBSS, fixed with 2% paraformaldehyde in PBS for 1 h on ice, and washed × 3 with PBS for flow cytometry (BD LSR II), as described^11,37^. The live cell gate was determined with FSC versus SSC and the number of GFP-positive cells, indicative of bacterial uptake, determined.

### Opsonophagocytosis

HL60 and DMF-differentiated dHL60 cells were counted and suspended at 3 × 10^5^ cells/ ml in opsonization buffer (HBSS with calcium and magnesium, 0.1% gelatin, 10% FBS), as described^9^. *P. aeruginosa* IATS06 was grown overnight in Hy-Soy medium to stationary phase, washed and resuspended in PBS to an OD of 0.3 corresponding to a concentration of 1.2 × 10^8^ CFU/ml. Bacteria were diluted in PBS to a working stock of 1.2 × 10^4^/ml. Ten uL of the working stock (i.e. 1020 CFU) were added to wells in a 96-well microtiter plate in the presence or absence of 10 µl of baby rabbit complement (BRC) (Pel Freez Biologicals, Lot# 31,061-3) and 10 µl of heat-inactivated rabbit antisera raised against heat-killed PA IATS 06 or opsonization buffer. Bacterial opsonization was allowed to proceed in the microtiter wells for 15 min at 37 °C, after which time, 1.2 × 10^5 ^HL60 cells in 40 µl were added to the wells (MOI = 1000 HL60 cells: 1 bacteria). The microtiter plates were then incubated for 45 min at 37 °C on a shaking platform, after which 10 µl from each well was plated onto Hy-Soy agar, incubated overnight at 30 °C, and colony counts determined.

### Statistical analyses

Experimental data were expressed as mean ± standard error values. Pairwise comparisons of groups were performed utilizing two-tailed unequal variance Student’s t-test. Multiple groups were analyzed using one-way ANOVA. To probe data sets for dose–response relationships, the Spearman rank correlation was applied. Statistical significance was set at *p* < 0.05.

## Results

### NEU activity in unstimulated human PMNs

**Figure 1:**
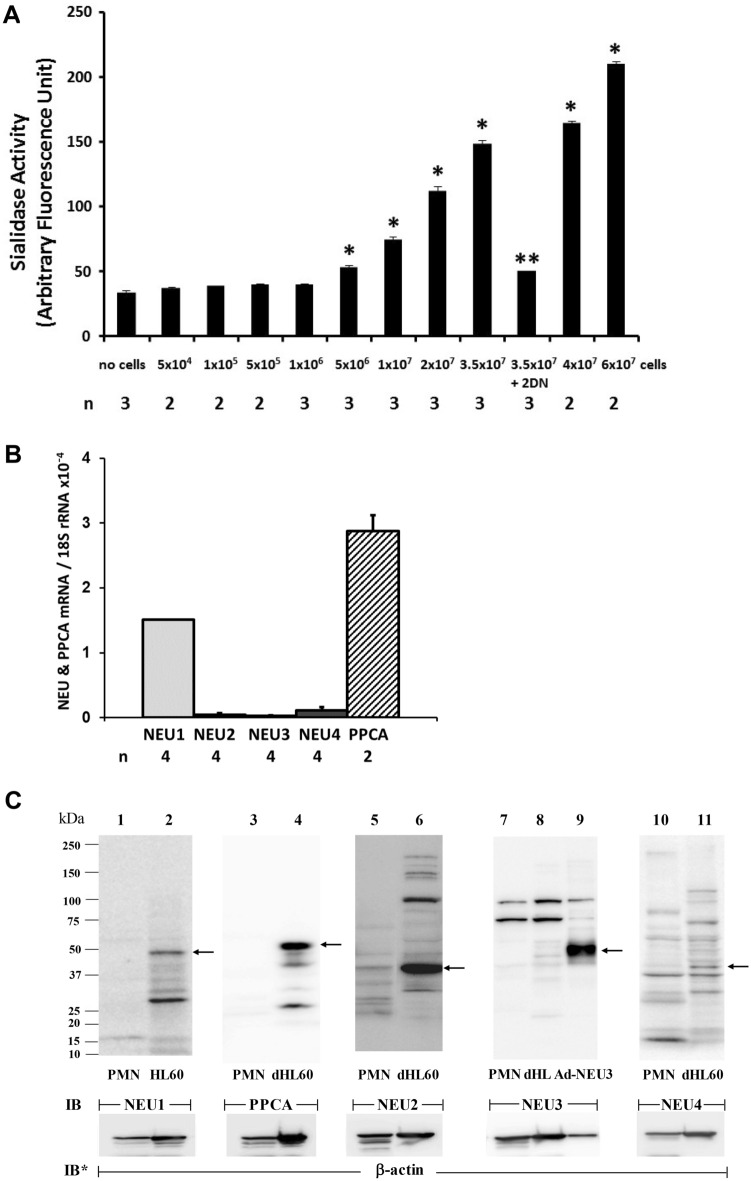
Human PMN NEU Activity and NEU and PPCA Expression. (**A**) Increasing cell numbers of unstimulated PMNs were assayed in the presence and absence of 2DN (500 µg/ml), for NEU activity for the 4-MU-NANA substrate. Vertical bars represent mean ± SE NEU activity expressed as arbitrary fluorescence units. *, significantly increased compared with NEU activity associated with the cell-free control at *p* < 0.05. **, significantly decreased compared with NEU activity of 3.5 × 10^7^ PMNs in the absence of 2DN at *p* < 0.05. (**B**) Total RNA isolated from PMNs was processed for qRT-PCR for NEU1, NEU2, NEU3, NEU4, and PPCA mRNA levels. The mRNA levels for each NEU and PPCA were normalized to the 18S rRNA internal control. Each vertical bar represents mean ± SE normalized mRNA levels. (**C**) PMNs were lysed and the lysates, at 50 µg total cellular protein/lane, were processed for NEU1 (lanes 1–2), PPCA (lanes 3–4), NEU2 (lanes 5–6), NEU3 (lanes 7–9), and NEU4 (lanes 10–11) immunoblotting. Lysates from HL60 cells were used as a positive control for NEU1 (lane 2), whereas lysates from dHL60 cells were used as positive controls for PPCA (lane 4), NEU2 (lane 6), NEU3 (lane 8), and NEU4 (lane 11). Lysates from Ad-NEU3 infected A549 cells were also used as a NEU3 positive control (lane 9). To control for protein loading and transfer, blots were stripped and reprobed for β-actin. IB, immunoblot; IB*, immunoblot after stripping. MW in kDa indicated on left. Arrows on right indicate bands with the anticipated gel mobilities of the proteins of interest. The data generated in each panel represents experiments performed on ≥ 2 independent occasions. Cropped immunoblot images are shown.

We previously reported endogenous NEU activity in mature human PMNs^31^. In the current studies, increasing PMN cell numbers were assayed for NEU activity for the fluorogenic substrate, 4-MU-NANA (Fig. 1A). PMNs at ≥ 5 × 10^6 ^cells dose-dependently expressed increasing NEU activity. The application of the Spearman rank correlation revealed a dose–response relationship between PMN cell number and NEU activity. The PMN NEU activity was almost completely inhibited by the broad-spectrum, competitive NEU inhibitor, 2DN (Fig. 1A). We asked which one or more of the 4 known NEU(s) might be operative.

### NEU and PPCA gene expression in human PMNs

qRT-PCR was used to detect transcripts for NEU1, -2, -3, and -4, and PPCA (Fig. 1B). Each NEU/PPCA mRNA was normalized to 18S rRNA. Of the 4 NEUs, their relative mRNA abundance was NEU1 > NEU4 > NEU2 ≈ NEU3. NEU1 mRNA was expressed at levels ≥ 14-fold greater than mRNA levels for each of the other 3 NEUs (Fig. 1B). However, PPCA mRNA was expressed at ~ twofold higher levels than was NEU1 itself (Fig. 1B).

### NEU and PPCA protein expression in human PMNs

We then asked whether one or more NEUs and/or PPCA might be expressed in mature human PMNs at the protein level. Unstimulated PMNs were lysed and the lysates, at 50 µg total cellular protein/lane, were processed for NEU1, PPCA, NEU2, NEU3, and NEU4 immunoblotting (Fig. 1C). Since NEU1 protein levels were highest in nondifferentiated HL60 cells (see Fig. 5A), lysates from these same cells were used as simultaneous, positive gel-mobility controls for NEU1, whereas lysates of dHL60 cells were used as positive controls for PPCA, NEU2, NEU3, and NEU4 (see Fig. 5C, E, G and I). NEU1, PPCA, NEU3, and NEU4 proteins were not detected in PMN lysates (Fig. 1C, lanes 1, 3, 7, and 10), whereas NEU2 protein was (Fig. 1C, lane 5). As an additional positive control for NEU3, A549 cells were infected with adenovirus encoding for human NEU3 (Ad-NEU3) as described^43^, and the NEU3- overexpressing cells were lysed and the lysates processed for NEU3 immunoblotting (Fig. 1C, lane 9). In each case, a NEU3-reactive band that displayed comparable gel mobilities was not detected. These combined data indicate that at the protein level, mature human PMNs do not express NEU1, its chaperone, PPCA, NEU3, or NEU4, but do express NEU2. For Fig. 1C, complete representative blots are displayed in Fig. S1C.

### NEU and PPCA protein expression in PMNs undergoing apoptosis

**Figure 2:**
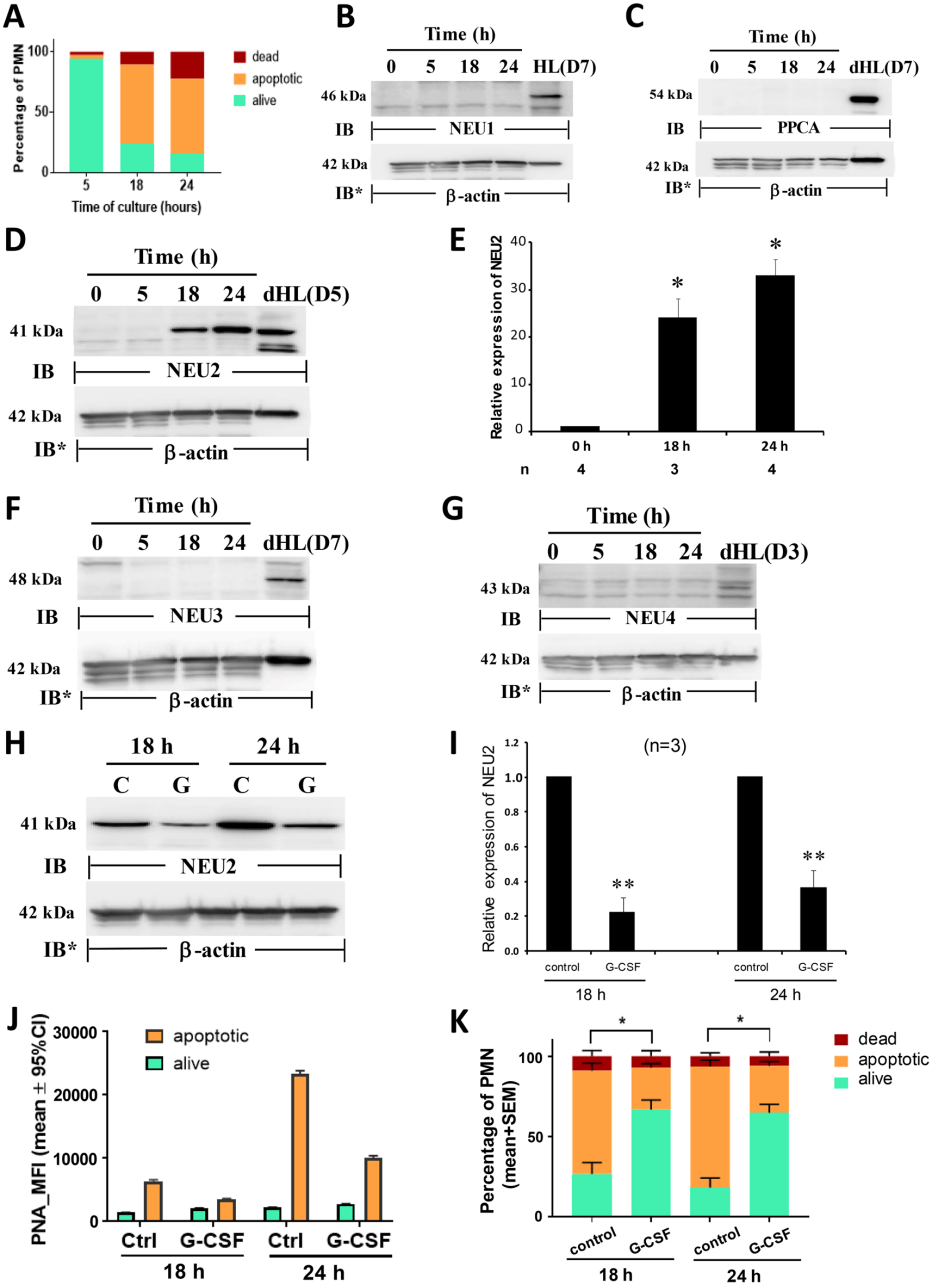
NEU2 Protein Expression in Proapoptotic PMNs. Purified human PMNs were cultured in RPMI 1640 with 10% FBS for 5, 18 or 24 h, after which the cells were assayed for viability and apoptosis (**A**) or lysed and the lysates, at 50 µg total cellular protein/lane, were processed for NEU1 (**B**), PPCA (**C**), NEU2 (**D**), NEU3 (**F**), and NEU4 (**G**) immunoblotting. To control for protein loading and transfer, blots were stripped and reprobed for β-actin. IB, immunoblot; IB*, immunoblot after stripping. MW in kDa indicated on left. (**E**) densitometric analyses of the blots in (**D**). Vertical bars represent mean ± SE NEU2 signal normalized to β-actin signal in the same lane on the same stripped and reprobed blot. *, increased normalized NEU2 signal compared to that seen at time 0. (**H**) PMNs were cultured for 18 h and 24 h, in the presence of human rG-CSF 10 ng/ml or medium alone, after which the cells were lysed and the lysates, at 50 µg total cellular protein/lane, were processed for NEU2 immunoblotting. To control for protein loading and transfer, blots were stripped and reprobed for β-actin. IB, immunoblot; IB* immunoblot after stripping. MW in kDa is indicated on the left. (**I**) Densitometric analyses of the blots in H. Vertical bars represent mean ± SE NEU2 signal normalized to β-actin signal in the same lane on the same stripped and reprobed blot. **, decreased normalized NEU2 signal compared to that seen in the absence of G-CSF treatment at *p* < 0.05. (**J**) PMNs were cultured for 18 h or 24 h in the presence of human rG-CSF or medium alone, after which they were subjected to flow cytometry to determine their viability or whether they had undergone apoptosis and PNA lectin flow cytometry to assess their surface desialylation. Vertical bars represent mean PNA MFI. (K) PMNs cultured for 18 h and 24 h, in the presence of G-CSF or medium alone, were assayed for viability and apoptosis. The data generated in each panel represents experiments performed on ≥ 2 independent occasions. Cropped immunoblot images are shown.

Mature PMNs were cultured in RPMI 1640 with 10% FBS for increasing times, after which they were assayed for apoptosis (Fig. 2A). As anticipated, PMN apoptosis increased over the 24 h study period. PMNs cultured for 5 h, 18 h, and 24 h, displayed 3.3%, 65.3%, and 62.0% apoptosis, respectively (Fig. 2A). Aliquots of these same cells were lysed and the lysates processed for NEU1, PPCA, NEU2, NEU3, and NEU4, immunoblotting (Fig. 2B–G). Over the 24 h study period, NEU2 protein expression dramatically increased while NEU1, 3, and 4 and PPCA protein expression were not detected. NEU2 protein expression increased > 25.0-fold at 18 h and > 30.0-fold at 24 h compared to that seen at 0 h (Fig. 2E). Since time-dependent, increasing PMN apoptosis was temporally coincident with increasing NEU2 protein expression, we asked whether these 2 cellular processes might be causally linked. Prior treatment with G-CSF, a cytokine that reportedly extends PMN survival^52^, protected against increases in NEU2 protein expression (Fig. 2H–I). The introduction of G-CSF decreased NEU2 protein expression at 18 h by ~ 80%, and at 24 h, by > 60%, each compared to their simultaneous controls (Fig. 2I). NEU2 was the only NEU protein detected in PMNs (Figs. 1C and 2B–G). As an indirect measure of NEU2 catalytic activity in proapoptotic PMNs, PMN surface desialylaion was measured, using PNA lectin flow cytometry (Fig. 2J). In the absence or presence of G-CSF treatment, the PNA signal i.e., desialylation for viable cells, remained unchanged over the 24 h time period. At 18 h and 24 h, independent of G-CSF treatment, apoptotic cells consistently displayed greater PNA signal/desialylation then did viable cells. Temporally coincident with the increasing NEU2 protein expression in proapoptotic PMNs (Fig. 2D), PNA signal/desialylation similarly increased. From 18 to 24 h, PNA binding to apoptotic cells increased 3.7-fold. Just as G-CSF treatment prevented increased NEU2 protein expression in these same cells (Fig. 2H–I), it similarly protected against their desialylation by 46.1% at 18 h, and by 57.3% at 24 h (Fig. 2J). Finally, we asked whether the same G-CSF treatment of PMNs that diminished their NEU2 protein expression and desialylation might also protect against their apoptosis (Fig. 2K). G-CSF dramatically reduced PMN apoptosis at both 18 h and 24 h. At 18 h, G-CSF reduced PMN apoptosis by ~ 60%, and at 24 h, by > 60%, each compared to their simultaneous controls. Taken together, PMNs cultured in vitro display increased NEU2 protein expression, surface desialylation, and apoptosis, and G-CSF protects PMNs against all 3 of these changes. For Fig. 2B–D, F–H, complete representative blots are displayed in Fig. S2B, S2C, S2D, S2F, S2G, and S2H.

### NEU activity in HL60 and dHL60 cells

**Figure 3:**
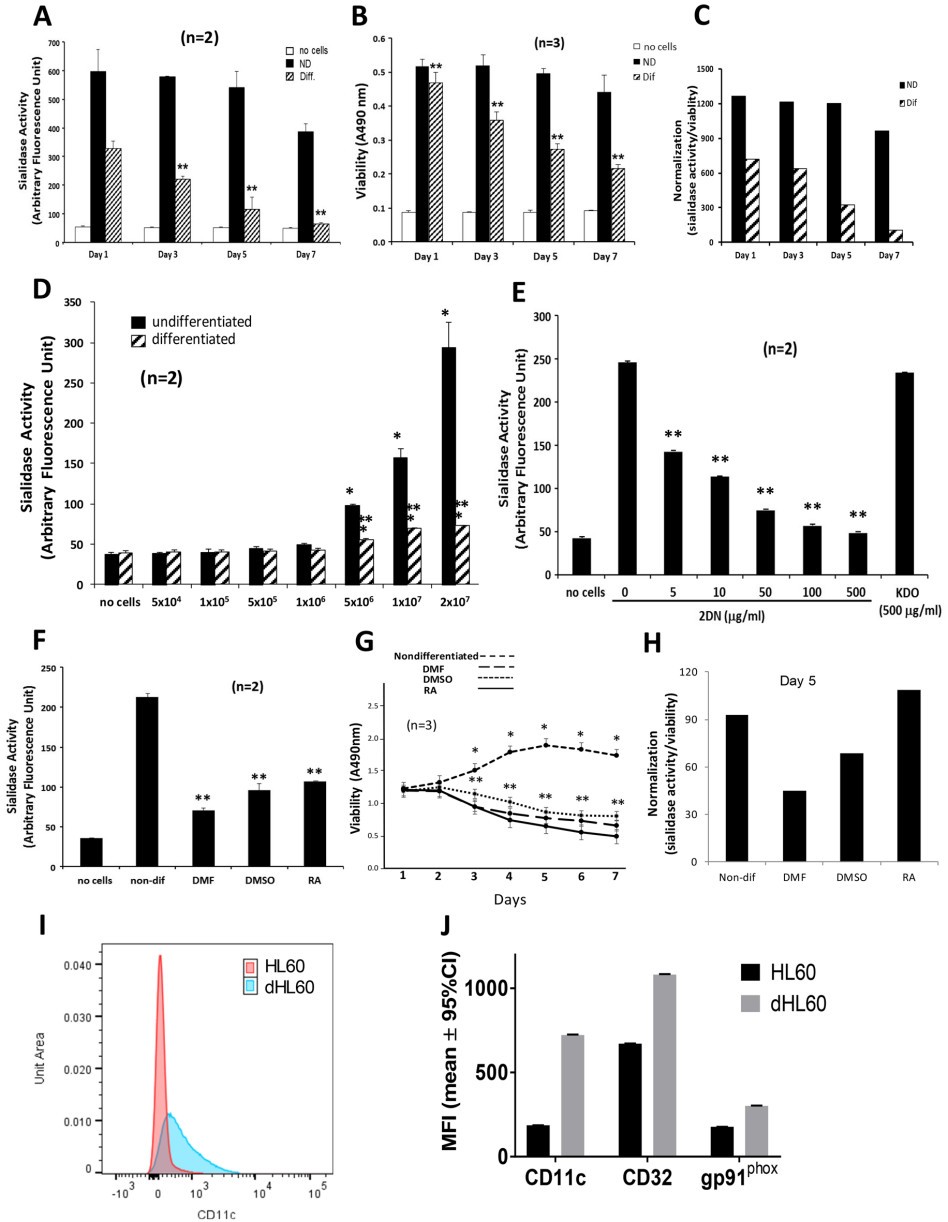
Total NEU Activity in HL60 and dHL60 Cells. (**A**, **B**) HL60 cells (2.5 × 10^7^ cells/assay) were cultured for increasing times in the presence of DMF or medium alone and assayed for NEU activity for the fluorogenic substrate, 4-MU-NANA (**A**) and cell viability (**B**). (**C**) Sialidase activity in (**A**) was normalized to cell viability in (**B**). (**D**) Increasing equivalent cell numbers of HL60 and dHL60 cells were assayed for NEU activity. (**E**) HL60 cells (2 × 10^7^ cells/sample) in the presence of increasing concentrations of the NEU inhibitor, 2DN, or the KDO negative control, were assayed for NEU activity. (**F**, **G**) HL60 cells were cultured for 7 days in the presence of DMF, DMSO, RA, or medium alone. Equivalent HL60 and dHL60 cell numbers (2 × 10^7^ cells/sample) were assayed for NEU activity (**F**) and cell viability (**G**). (**H**) NEU activity in (**F**) was normalized to cell viability at 5 days in (**G**). (**A**–**F**, **H**) Vertical bars or symbols represent mean ± SE NEU activity expressed as arbitrary fluorescence units (**A**, **D**, **E**, and **F**) or mean ± SE cell viability expressed as cellular uptake of MTT (**B** and **G**). *, significantly increased compared with NEU activity or viability in equivalent numbers of dHL60 cells at *p* < 0.05. **, significantly decreased compared with NEU activity or cell viability in nondifferentiated HL60 cells (**A**, **B**, **D**, **F**, **G**) or HL60 cells in the absence of 2DN (**E**) at *p* < 0.05. (**I**, **J**) HL60 and DMF-differentiated HL60 cells were stained with fluoroprobe-labeled anti-CD11c (**I**, **J**), anti-CD32 (**J**), and anti-gp91^phox^ (**J**) antibodies, along with 7-AAD to exclude dead cells. (**I**) Histograms of CD11c expression in HL60 and dHL60 cells and (**J**) the mean fluorescence intensity (MFI) for each fluoroprobe for live cells. *, significantly increased MFI compared with the MFI for the same fluoroprobe for nondifferentiated HL60 cells. The data generated in each panel represents experiments performed on ≥ 2 independent occasions.

Endogenous NEU activity has been detected in PMNs^31^ and HL60 cells^5^, and the HL60 leukemic cell line can be differentiated in cell culture towards PMNs^9^. Differentiated HL60 (dHL60) cells are often used as a surrogate for human PMNs. We asked how PMN NEU activity might compare to NEU activity in HL60 cells differentiated towards the PMN phenotype, and whether the differentiation process might influence HL60 NEU activity. HL60 cells cultured for increasing times in the presence of DMF or medium alone were assayed for NEU activity for the fluorogenic substrate, 4-MU-NANA (Fig. 3A). On days 1, 3, 5, and 7 of differentiation, mean NEU activity in the dHL60 cells was reduced by 52. 2%, 61.0%, 84.1%, and 94.3% respectively, compared to that detected in nondifferentiated HL60 cells cultured for the same time periods. As DMF exposure time increased, NEU activity decreased. In contrast, NEU activity in nondifferentiated HL60 cells was sustained through day 5 with a reduction of 44.5% observed on day 7. Therefore, HL60 cells differentiated into PMN-like cells and mature PMNs displayed comparable levels of NEU activity. Since cellular differentiation entails growth arrest and programmed cell death^9^, we studied the viability of HL60 cells undergoing differentiation (Fig. 3B). After 1, 3, 5, and 7 day(s) of DMF exposure, mean viability for the dHL60 cells decreased 9.1%, 31.2%, 44.8%, and 50.8%, respectively (Fig. 3B). In contrast, viability in HL60 cells did not change. When NEU activity was normalized to cell viability, after 1, 3, 5, and 7 day(s) of DMF exposure, 17.4%, 51.1%, 53.3%, and 53.9% of these decreases in NEU activity, respectively, could be ascribed to loss of dHL60 cell viability (Fig. 3C). Increasing total cell numbers of HL60 and dHL60 cells were assayed for NEU activity (Fig. 3D). The HL60 cells at ≥ 5 × 10^6^ cells dose-dependently expressed increased NEU activity. The application of the Spearman rank correlation revealed a dose–response relationship between HL60 cell number and NEU activity. Increasing identical numbers of dHL60 cells were associated with significant but only modest increases in NEU activity. At equivalent cell numbers, the NEU activity associated with HL60 cells could be > fourfold higher than that associated with dHL60 cells. The NEU activity of 2.0 × 10^7^ HL60 cells was dose-dependently inhibited by the broad-spectrum, competitive NEU inhibitor, 2DN, but not by the KDO negative control (Fig. 3E). The application of the Spearman rank correlation revealed a dose–response relationship between 2DN concentration and inhibition of HL60 NEU activity. To confirm that the differentiation process decreased NEU catalytic activity, HL60 cells were cultured for 7 days in the presence of 3 distinct differentiation agents, DMF, DMSO, or RA, or medium alone, and assayed for NEU activity (Fig. 3F). Each of the 3 differentiation agents dramatically reduced NEU activity by 84.3, 68.2%, and 65.8%, respectively, compared to the simultaneous, nondifferentiated controls. Under identical experimental conditions, the viability of nondifferentiated HL60 cells were maintained, and with ongoing cell proliferation, cell number actually increased at ≥ 3 days compared to that seen on day 1 (Fig. 3G). In contrast, the viability of dHL60 cells in response to DMF, DMSO, or RA, each decreased at ≥ 3 days compared to the simultaneous, nondifferentiated controls. By 7 days, the viability of dHL60 cells decreased by 54–77% compared to that seen in the simultaneous, nondifferentiated controls. This loss of viability (Fig. 3B, G) was compatible with, and even indicative of the differentiation process^9^. Again, NEU activity was normalized to cell viability (Fig. 3H). These combined data indicate that the differentiation process is consistently associated with both diminished NEU activity and cell viability. However, only a portion of the reduction in NEU activity could be ascribed to loss of cell viability. To validate the differentiation process, HL60 cells exposed to DMF for 5 days were assessed for expression of three myeloid maturation markers, CD11c, CD32, and gp91^phox^, as described^50,51^ (Fig. 3I, J). As anticipated, exposure to DMF increased expression of each differentiation marker in dHL60 cells compared to that seen in HL60 cells.

### NEU and PPCA gene expression during HL60 cell differentiation

**Figure 4:**
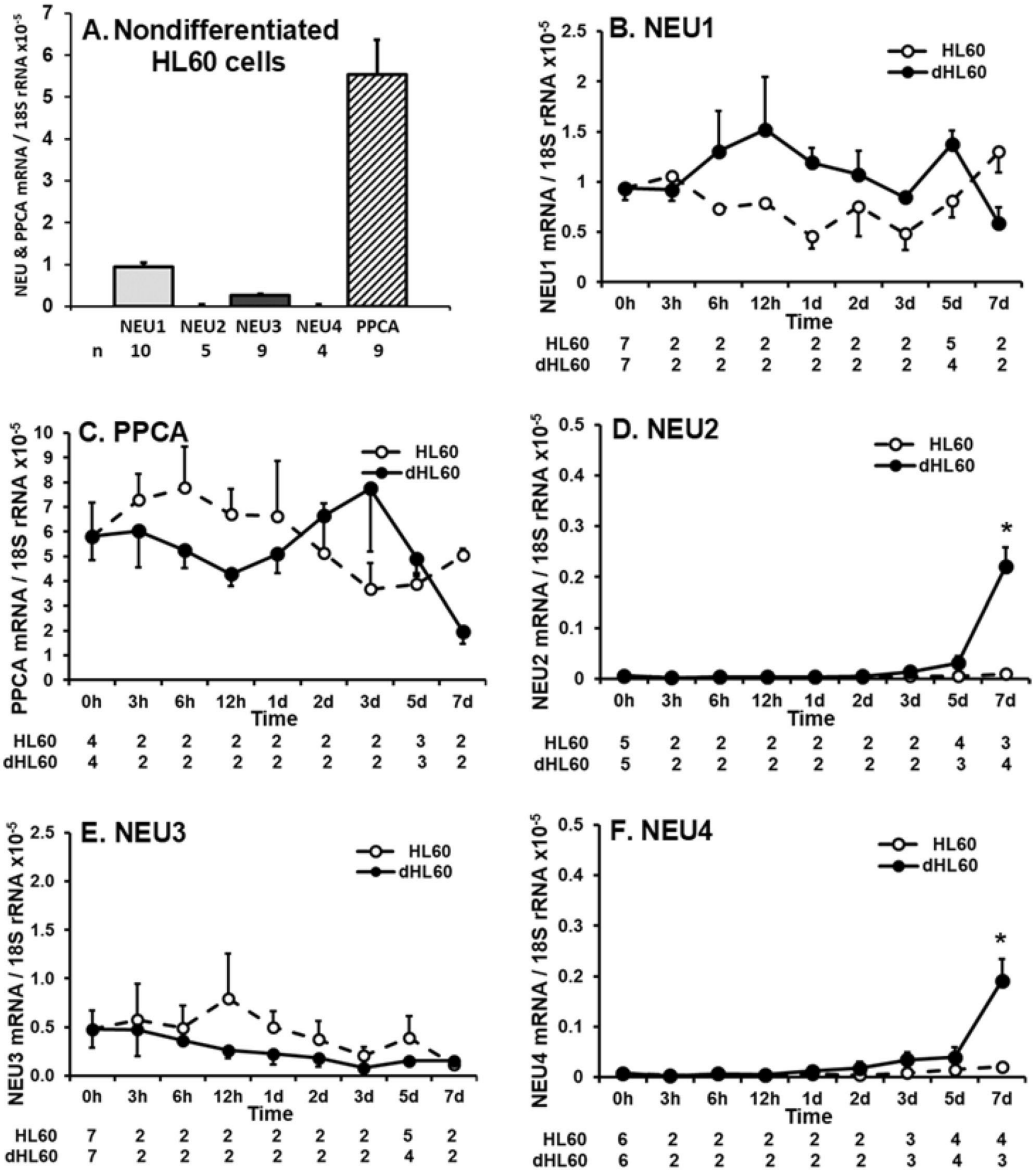
NEU and PPCA Gene Expression During HL60 Cell Differentiation. (**A**) Total RNA isolated from nondifferentiated HL60 cells was processed for qRT-PCR for mRNA levels for NEU1-4 and PPCA, and normalized to the 18S rRNA control. (**B**–**F**) Total RNA isolated from HL60 cells cultured for increasing times in the presence of DMF or medium alone was processed for qRT-PCR to quantify mRNA levels for NEU1 (**B**), PPCA (**C**), NEU2 (**D**), NEU3 (**E**), and NEU4 (**F**), and each normalized to the 18S rRNA control. The n for each data point is indicated below each vertical bar (**A**) or time point (**B**–**F**). Each vertical bar (**A**) or symbol (**B**–**F**) represents mean ± SE normalized mRNA levels. The data generated in each panel represents experiments performed on ≥ 2 independent occasions. *, increased normalized NEU2/4 mRNA expression compared to nondifferentiated cells at *p* < 0.05.

HL60 cells express modest levels of NEU activity for the 4-MU-NANA substrate (Fig. 3A, D, F). We asked whether one or more of the 4 reported mammalian NEUs might be expressed in these same cells at the mRNA level. In HL60 cells, qRT-PCR was used to quantify transcripts for NEU1-4, each normalized to 18S rRNA (Fig. 4A). NEU1 mRNA was expressed at the highest levels, 3.7-fold higher than NEU3 mRNA, which was expressed at the second highest levels. NEU2 and -4 mRNAs were essentially undetectable, with mRNA levels ~ 200-fold less than NEU1 levels. Since NEU1 was expressed in HL60 cells and its activity absolutely requires its association with the chaperone/transport protein, PPCA^40,41^, qRT-PCR was used to assay for PPCA transcripts (Fig. 4A). PPCA mRNA expression was detected at levels 5.8-fold higher than NEU1. These results were similar to but distinct from what was found in mature PMNs (Fig. 1B) where the relative mRNA expression levels were PPCA > NEU1 > NEU4 > NEU2 ≈ NEU3. For both the PMN and HL60 cell studies, all qRT-PCR data were normalized to the 18S rRNA internal control. Each data point was expressed as the ratio of NEU/PPCA amplification target to the 18S rRNA. With similar amplification rates across these targets, the normalized data for PMNs in Fig. 1B are directly comparable to the normalized data for HL60 cells in Fig. 4A (see Fig. S12). Differentiation of HL60 cells profoundly diminishes their total NEU activity (Fig. 3A, F). We asked whether expression of NEU1-4 and/or PPCA might change during differentiation. HL60 cells were cultured for increasing times in the presence of DMF or medium alone, and RNA harvested and processed for qRT-PCR for NEU1 (Fig. 4B), PPCA (Fig. 4C), NEU2 (Fig. 4D), NEU3 (Fig. 4E), and NEU4 (Fig. 4F) mRNA levels. Although no clear, consistent trend for changes in NEU1 and PPCA mRNA expression in dHL60 cells could be established, each displayed both an early (12 h and 3 h, respectively) and later (5 and 3 days, respectively) increase, but by day 7, both decreased (Fig. 4B, C). However, these changes did not achieve statistical significance. Although neither NEU2 nor NEU4 mRNA expression could be detected in nondifferentiated HL60 cells (Fig. 4A), expression of each of these two genes was upregulated on day 7 (Fig. 4D, F). NEU3 mRNA expression remained unchanged throughout the 7-day period (Fig. 4E).

### NEU1-4 and PPCA protein expression during HL60 cell differentiation

**Figure 5:**
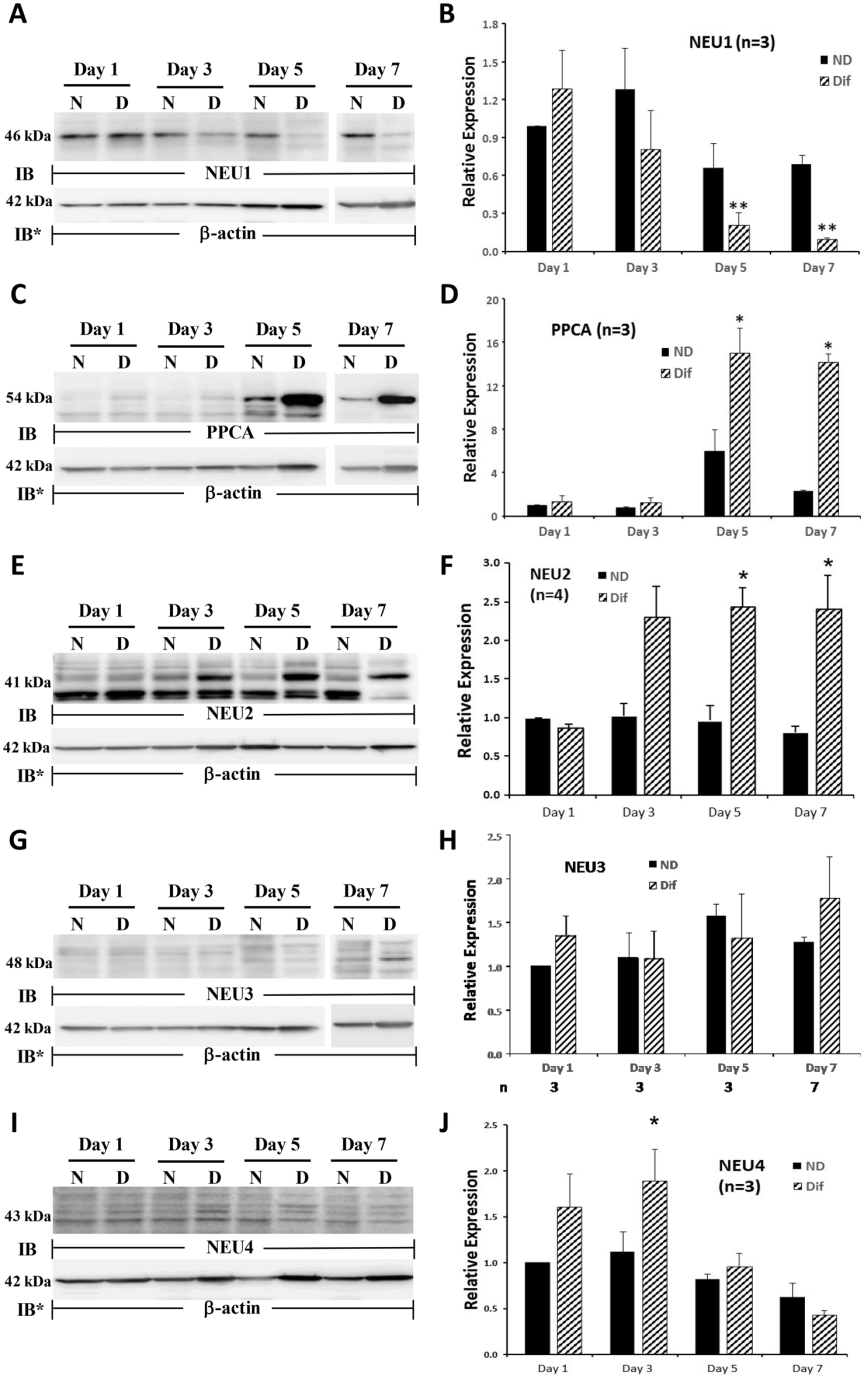
NEU and PPCA Protein Expression During HL60 Cell Differentiation. HL60 cells were cultured for 1, 3, 5 or 7 days in the presence of DMF or medium alone, lysed, and the lysates, at 50 µg total cellular protein/lane, were processed for NEU1 (**A**), PPCA (**C**), NEU2 (**E**), NEU3 (**G**), and NEU4 (**I**) immunoblotting. (**A**, **C**, **E**, **G**, and **I**) to control for protein loading and transfer, blots were stripped and reprobed for β- actin. IB, immunoblot; IB*, immunoblot after stripping. MW in kDa is indicated on the left. (**B**, **D**, **F**, **H**, and **J**) densitometric analyses of the blots in A, C, E, G, and I, respectively. Vertical bars represent mean ± SE NEU1, PPCA, NEU2, NEU3, or NEU4 signal normalized to β-actin signal in the same lane on the same stripped and reprobed blot. *, increased normalized PPCA, NEU2, or NEU4 signal in dHL60 cells compared to nondifferentiated cells at *p* < 0.05. **, decreased normalized NEU1 signal in dHL60 cells compared to nondifferentiated cells at *p* < 0.05. Each blot is representative of 3–7 independent experiments. Cropped immunoblot images are shown.

We then asked whether NEU1, PPCA, NEU2, NEU3, and/or NEU4 proteins were expressed in HL60 cells, and if so, whether their expression might be altered during differentiation (Fig. 5). HL60 cells were cultured for 1, 3, 5, or 7 days in the presence of DMF or medium alone, lysed, and the lysates, at 50 µg total cellular protein/lane, were processed for NEU1 (Fig. 5A, B), PPCA (Fig. 5C, D), NEU2 (Fig. 5E, F), NEU3 (Fig. 5G, H), and NEU4 (Fig. 5I, J) quantitative immunoblotting. In HL60 cells, NEU1 and NEU2 proteins were clearly expressed whereas PPCA, NEU3, and NEU4 protein expression was modest or undetectable. In HL60 cells cultured < 3 days in the presence of DMF, no changes in NEU1, PPCA, NEU2, NEU3, or NEU4 protein expression could be detected, compared to the simultaneous nondifferentiated controls (Fig. 5A–J). In the dHL60 cells, on days 5 and 7, NEU1 protein expression decreased > 90% compared to its expression in nondifferentiated cells (Fig. 5A, B). Of note, even in the nondifferentiated cells, NEU1 expression by days 5 and 7 was diminished by ~ 30% compared to NEU1 expression on day 1. In contrast, over the same 7-day period, PPCA protein expression increased > tenfold (Fig. 5C, D). On day 5, PPCA expression in the HL60 cells was elevated almost sixfold compared to its expression on day 1. The anti-PPCA antibody used in these experiments recognizes the 54 kDa precursor, but not its cleavage products. Over the same time period, NEU2 expression increased up to 5.8-fold (Fig. 5E, F). Elevated NEU2 expression was evident on days 3, 5, and 7. However, the increase on day 3 did not achieve statistical significance (*p* = 0.0603). Over the 7 day period, NEU3 protein expression in either HL60 or dHL60 cells did not change (Fig. 5G, H). NEU4 expression, on day 3, increased 1.7-fold, after which it returned to baseline on days 5 and 7 (Fig. 5I, J). For Fig. 5A, C, E, G and I, complete representative blots are displayed in Fig. S5A, S5C, S5E, S5G, and S5I. In summary, dramatic changes in NEU1, PPCA, NEU2, and NEU4 but not NEU3 protein expression were detected during the differentiation process. Based on these combined data, the regulation of NEU and PPCA protein expression does not appear to be controlled by their respective steady-state mRNA levels.

**Figure 6:**
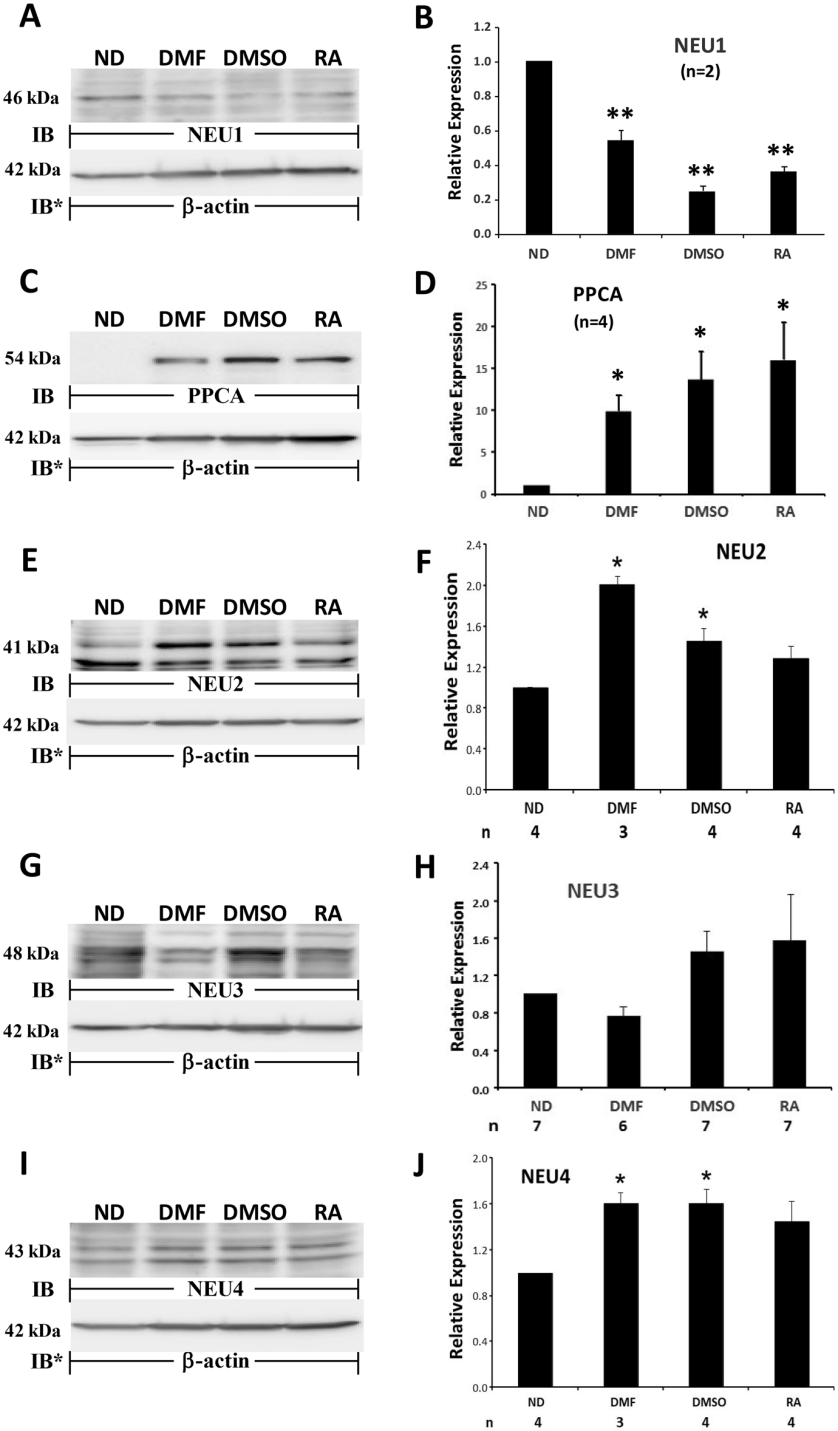
NEU and PPCA Protein Expression in Response to Multiple Differentiation Agents. HL60 cells were cultured for 7 days in the presence of DMF, DMSO, RA, or medium alone, lysed, and the lysates, at 50 µg total cellular protein/lane, were processed for NEU1 (**A**), PPCA (**C**), NEU2 (**E**), and NEU3 (**G**) immunoblotting. For the NEU4 immunoblots, cells were harvested on day 3 (**I**). (**A**, **C**, **E**, **G**, and **I**), to control for protein loading and transfer, blots were stripped and reprobed for β-actin. IB, immunoblot; IB*, immunoblot after stripping. MW in kDa is indicated on the left. (**B**, **D**, **F**, **H**, and **J**), densitometric analyses of the blots in (**A**, **C**, **E**, **G**, and **I**), respectively. n for each experimental group indicated in each panel (**B** and **D**) or under each vertical bar (**F**, **H**, and **J**). Vertical bars represent mean ± SE NEU1, PPCA, NEU2, NEU3, or NEU4 signal normalized to β-actin signal in the same lane on the same stripped and reprobed blot. *, increased normalized PPCA, NEU2, or NEU4 signal in dHL60 cells compared to HL60 cells at *p* < 0.05. **, decreased normalized NEU1 signal in dHL60 cells compared to HL60 cells at *p* < 0.05. Each blot is representative of 3–7 independent experiments. Cropped immunoblot images are shown.

To confirm that the differentiation process decreased NEU1 protein expression (Fig. 5A, B), and increased PPCA, NEU2, and NEU4 protein expression (Fig. 5C–F and I–J), but failed to influence NEU3 protein expression (Fig. 5G, H), HL60 cells were cultured for 7 days in the presence of DMF and 2 other distinct differentiation agents, DMSO, and RA, or medium alone, lysed, and the lysates, at 50 µg total cellular protein/lane, were processed for NEU1 (Fig. 6A, B), PPCA (Fig. 6C, D), NEU2 (Fig. 6E, F), and NEU3 (Fig. 6G, H) quantitative immunoblotting. For the NEU4 immunoblots, the cells were harvested on day 3 (Fig. 6I, J). Each of the 3 distinct differentiation agents, DMF, DMSO, and RA, reduced NEU1 protein expression 48.0%, 68.8% and 52.2%, respectively (Fig. 6A, B), and increased PPCA protein expression by 7.7-fold, 11.3-fold, and 12.6-fold, respectively, (Fig. 6C, D), in dHL60 cells compared to that seen in HL60 cells. NEU2 expression was increased 2.0-fold, 1.5-fold, and 1.3-fold, respectively (Fig. 6E, F), and at 3 days, NEU4 expression increased 1.6-fold, 1.6-fold, and 1.5-fold, respectively (Fig. 6I, J). For NEU2 and -4, the RA-induced increases did not achieve statistical significance. None of the 3 differentiation agents altered NEU3 protein expression (Fig. 6G, H). Although DMSO and RA each increased mean NEU3 protein expression, even with robust n’s (n = 7), neither increase achieved statistical significance (Fig. 6H). For Fig. 6A, C, E, G, and I, complete representative blots are displayed in Fig. S6A, S6C, S6E, S6G, and S6I.

### Sialylation changes in HL60 cells undergoing differentiation

**Figure 7:**
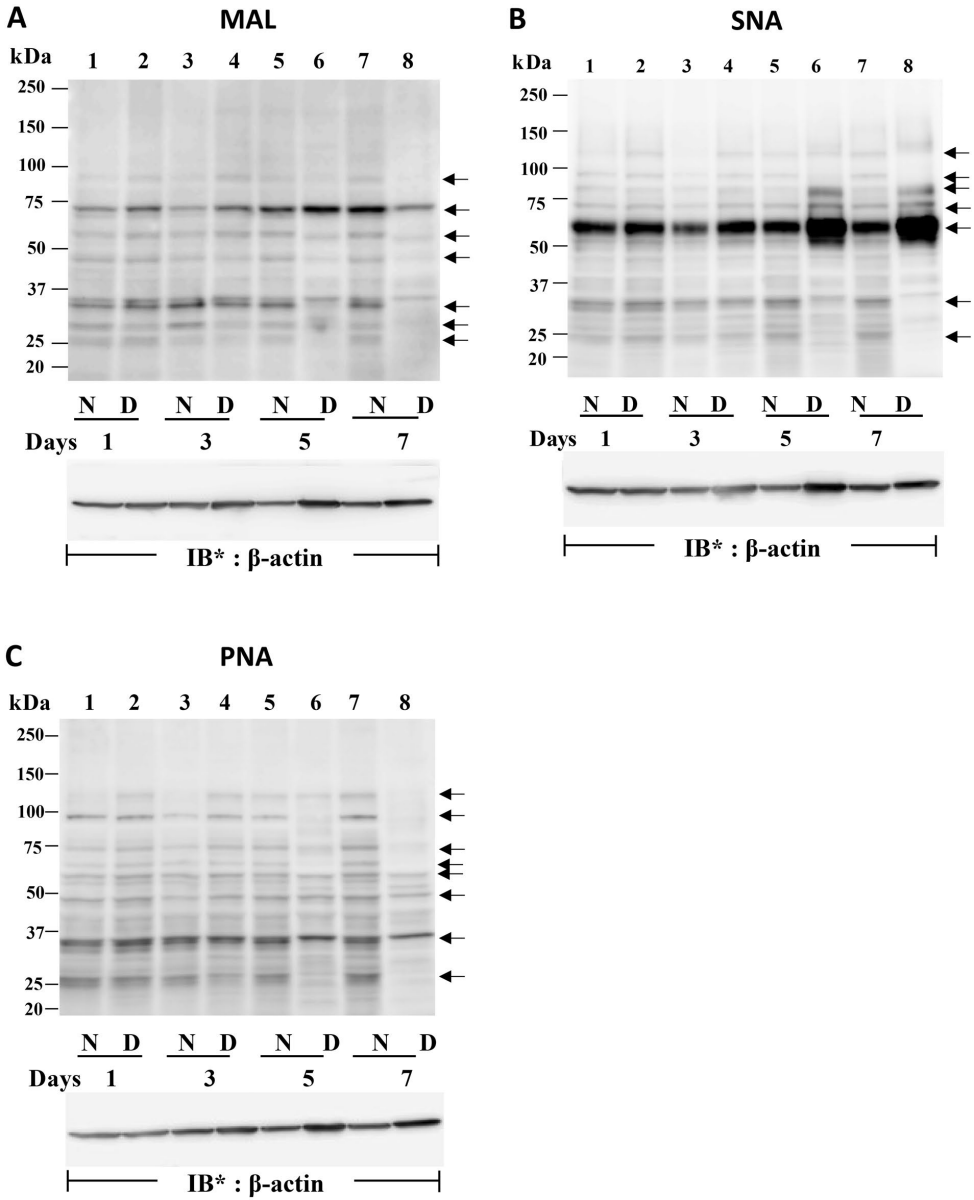
Changes in HL60 Cell Sialylation During Differentiation. (**A–C**) HL60 cells were cultured for 1, 3, 5, and 7 days in the presence of DMF or medium alone, lysed, and the lysates, at 50 µg total cellular protein/lane, were resolved by SDS-PAGE, transferred to PVDF, and probed with biotinylated MAL (**A**), SNA (**B**), or PNA (**C**), each followed by incubation with HRP-conjugated streptavidin and ECL reagents. To control for protein loading and transfer, blots were stripped and reprobed for β-actin. IB = immunoblot; IB* = immunoblot after stripping. MW in kDa indicated on left. Arrows on right indicate unidentified bands in which sialylation changes over time. Each lectin blot is representative of 3 independent experiments. Cropped lectin blot images are shown.

In the face of dramatic changes in NEU1 (Fig. 5A, B), PPCA (Fig. 5C, D), NEU2 (Fig. 5E, F), and NEU4 (Fig. 5I, J), and sustained levels of NEU3 (Fig. 5G, H) protein expression during HL60 cell differentiation, we tested whether temporally coincident changes in sialylation could be detected. First, we used fetuin and asialofetuin as positive and negative controls to validate the MAL, SNA, and PNA lectins (Figure S7D). As anticipated, MAL and SNA only recognized fetuin whereas PNA only bound to asialofetuin. HL60 cells were cultured for 1, 3, 5, and 7 days in the presence of DMF or medium alone, lysed, and the lysates, at 50 µg total cellular protein/lane, were processed for MAL (Fig. 7A), SNA (Fig. 7B), and PNA (Fig. 7C) lectin blotting. MAL-reactive bands, i.e. α2,3-linked SA residues, were detected in HL60 cells with gel mobilities compatible with approximate MW’s of 90 kDa, 72 kDa, 60 kDa, 46 kDa, 35 kDa, 28 kDa, and 25 kDa (Fig. 7A, lane 7). These same MAL-reactive bands each was reduced in MAL lectin blots of dHL60 cells (Fig. 7A, lane 8). Whether these decreases in α2,3-linked sialylation could be ascribed to the temporally coincident increase in NEU2 protein expression (Figs. 5E, F and 6E, F) was unclear. In contrast, SNA lectin blotting to probe for α2,6-linked sialylation revealed increased SNA-reactive signals in dHL60 cells with gel mobilities compatible with approximate MW’s of 120 kDa, 80 kDa, 72 kDa, and 60 kDa in dHL60 cells (Fig. 7B, lane 8) compared to that detected in HL60 cells (Fig. 7B, lane 7). In the dHL60 cells, the predominant 60 kDa SNA-reactive band increased by 2.3-fold compared to that seen in HL60 cells. On these same SNA lectin blots, a number of bands with approximate MWs of 90 kDa, 32 kDa, and 23 kDa, were diminished in intensity in dHL60 versus HL60 cells (Fig. 7B, lanes 8 vs 7). Finally, PNA lectin blotting which detects terminal galactose, often reflective of desialylation, revealed PNA-reactive signals with gel mobilities compatible with approximate MWs of 130 kDa, 98 kDa, 74 kDa, 65 kDa, 60 kDa, 47 kDa, 38 kDa, 34 kDa, and 25 kDa (Fig. 7C, lane 7), each of which decreased in dHL60 cells compared to the same bands detected in HL60 cells (Fig. 7C, lane 8). For Fig. 7A–C, complete representative β-actin blots are displayed in Fig. S7A, S7B, and S7C. To address whether these changes in sialylation of specific bands could be explained through altered NEU activity, HL60 cells were cultured for 7 days in the presence of DMF or medium alone, and in the presence or absence of 2DN, lysed, and the lysates processed for MAL, SNA, and PNA lectin blotting (Fig. S7E–G). The presence of 2DN did not alter patterns of sialylation, suggesting a NEU-independent process.

### MALDI ToF MS of sequentially labeled α2,3- and α2,6-linked sialyl N-glycans

**Figure 8:**
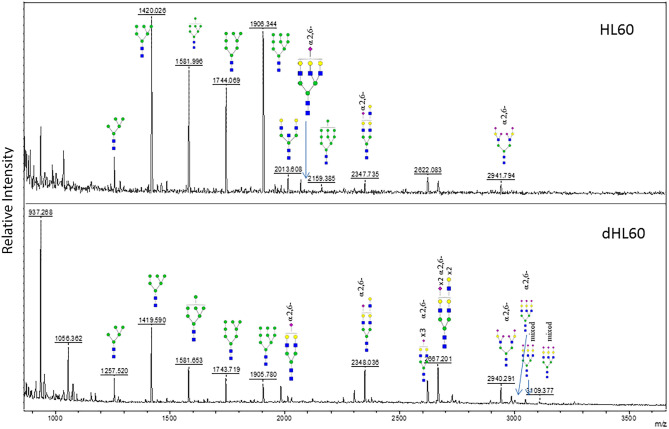
MALDI ToF MS of Sequentially Labeled α2,3- and α2,6-linked Sialyl N-glycans. N-glycans derived from HL60 (top panel) and dHL60 (bottom panel) cells were sequentially labeled by esterification and amidation of α2,3- and α2,6-linked SA, respectively. The α2,6-linked SA substituted N-glycans were dramatically increased in dHL60 cells. Cartoonized structures shown with the α2,6- label denote exclusive substitution with α2,6-linked SA whereas those denoted as mixed contain both α2,3- and α2,6-linked SA substitutions. Symbols represent the following monosaccharides: Blue square, GlcNAc; Green circle, Man; Yellow circle, Gal; Pink diamond, NeuAc. These data are representative of 2 independent experiments.

N-glycans from HL60 cells were analyzed by MALDI ToF MS. HL60 cells were cultured for 7 days in the presence of DMF or medium alone. Cellular protein was processed using solid phase chemical modification of SAs. The α2,6- and α2,3-linked SAs were modified sequentially via ethanol esterification and p-toluidine amidation, respectively. The α2,6 modification imparts a + 28 amu tag whilst that of the p-toluidine imparts a 89 amu tag^49^. Thus, modifications can be identified in the MALDI ToF MS spectra via these shifts relative to the theoretical unmodified SA. On the basis of these spectra, the presence of α2,6-linked SAs was clearly increased in dHL60 cells compared to that observed in nondifferentiated cells (Fig. 8). More specifically, the α2,6-linked SA substituted glycans at m/z 2348.03, 2667.20, 2940,29, 2986.52, 3047.41 and 3109.77 were substantially increased. While some N-glycans also contained α2,3-linked SAs, all those that were increased in abundance were predominantly increased in α2,6-linked SAs. These results were compatible with the decreased MAL-reactive bands (Fig. 7A) and increased SNA-reactive bands (Fig. 7B) found in lectin blotting of dHL60 cells.

### ST6GAL-1 and ST6GAL-2 gene expression during HL60 cell differentiation

**Figure 9:**
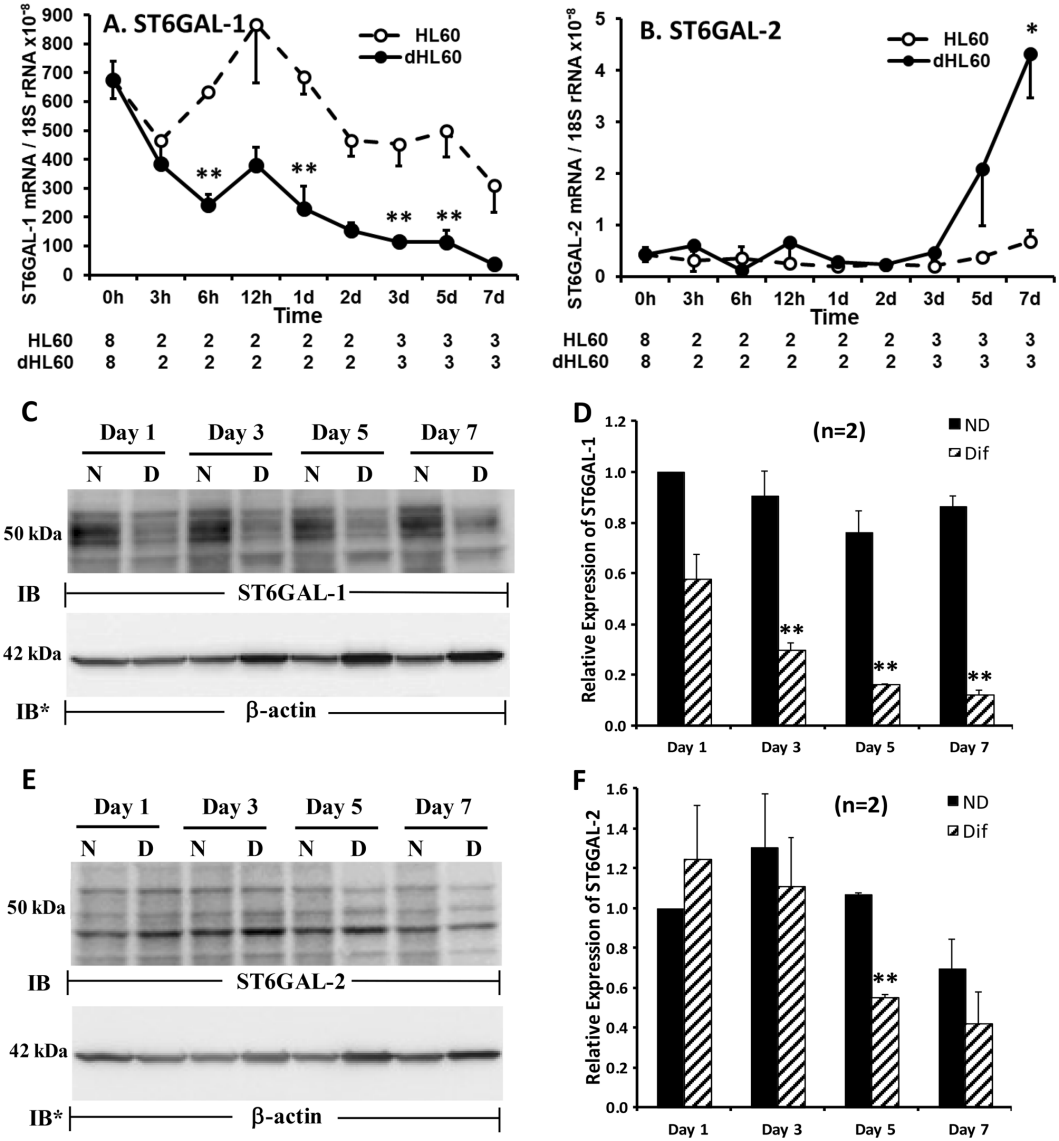
ST6GAL-1 and ST6GAL-2 Expression During HL60 Cell Differentiation. (**A**, **B**) Total RNA isolated from HL60 cells cultured for 0, 3, 6, and 12 h and 1, 2, 3, 5, and 7 days in the presence of DMF or medium alone was processed for qRT-PCR for human ST6GAL-1 (**A**) and ST6GAL-2 (**B**) mRNA levels. The mRNA levels for each ST was normalized to the 18S rRNA internal control. Each symbol represents mean ± SE normalized mRNA levels for each time point. n for each data point is indicated below each time point. (**C**, **E**) HL60 cells were cultured for 1, 3, 5, or 7 days in the presence of DMF or medium alone, lysed, and the lysates, at 50 µg total cellular protein/lane, were processed for ST6GAL-1 (**C**) and ST6GAL-2 (**E**) immunoblotting. To control for protein loading and transfer, blots were stripped and reprobed for β-actin. IB, immunoblot; IB*, immunoblot after stripping. MW in kDa is indicated on the left. (**D**, **F**) Densitometric analyses of the blots in (**C** and **E**), respectively. Vertical bars represent mean ± SE ST6GAL-1 and ST6GAL-2 signal normalized to β-actin signal in the same lane on the same stripped and reprobed blot. *, increased normalized ST6GAL-2 signal in dHL60 cells compared to HL60 cells at *p* < 0.05. **, decreased normalized ST6GAL-1 or ST6GAL-2 signal in dHL60 cells compared to HL60 cells at *p* < 0.05. The data generated in each panel represents experiments performed on ≥ 2 independent occasions.

In dHL60 cells, selected SNA lectin-reactive signals, i.e. α2,6-linked sialylation, dramatically increased (Fig. 7B) whereas selected PNA lectin-reactive signals decreased (Fig. 7C), compared to these same changes seen in nondifferentiated HL60 cells. While SA can be tethered to either galactose or N-acetylgalactosamine, PNA reportedly displays a relative affinity for galactose that is > 100-fold greater than that seen for N-acetylgalactosamine^53^. Taken together, these temporally coincident, reciprocal changes of increased α2,6-linked sialylation and decreased PNA binding to galactose are compatible with increased sialylation of galactose residues in α2,6-linkage. We asked whether either or both of the two STs known to transfer SA to galactose in α2,6-linkage, ST6GAL-1 and ST6GAL-2^24,25^, might be expressed in HL60 cells, and if so, whether their expression might change with differentiation. In HL60 cells, qRT-PCR was used to quantify ST6GAL-1 and -2 transcripts, which were normalized to 18S rRNA levels (Fig. 9A, B). In nondifferentiated HL60 cells, ST6GAL-1 mRNA was expressed at ~ 1600-fold higher levels than was ST6GAL-2 mRNA. In dHL60 cells, ST6GAL-1 mRNA levels decreased within 6 h of DMF treatment and continued to decrease over the 7-day study period compared to that detected in HL60 cells (Fig. 9A). In contrast, the modest basal levels of ST6GAL-2 mRNA only began to increase in dHL60 cells at 5 days of DMF treatment, and only achieved statistical significance at day 7, compared to the simultaneous nondifferentiated HL60 cells (Fig. 9B).

### ST6GAL-1 and ST6GAL-2 protein expression during HL60 cell differentiation

Over the 7-day differentiation period, ST6GAL-1 mRNA decreased (Fig. 9A), while ST6GAL-2 mRNA belatedly increased (Fig. 9B). We asked, whether during differentiation, ST6GAL-1 or -2 proteins might also change. ST6GAL-1 protein expression, in parallel with its mRNA expression, was dramatically and time-dependently reduced in dHL60 versus HL60 cells (Fig. 9C, D). In contrast, while ST6GAL-2 mRNA remained unchanged until > 5 days of differentiation (Fig. 9B), ST6GAL-2 protein expression decreased (Fig. 9E, F). These combined data are compatible with transcriptional regulation for ST6GAL-1 but not ST6GAL-2. For Fig. 9C, E, complete representative blots are displayed in Figures S9C and S9E. These data do not explain the increased α2,6-linked sialylation evident during HL60 differentiation (Fig. 7B). It is conceivable that the increase in α2,6-linked sialylation seen with differentiation could be explained through increased activity of one or more of the 6 STs known to transfer SA in α2,6- linkage to N-acetylgalactosamine^24,25^. However, prior broad-spectrum ST inhibition with CMP^27^ failed to prevent the differentiation-induced increases in α2,6-linked sialylation (Fig. S9G).

### Adhesion of HL60 and dHL60 cells to HBME monolayers

**Figure 10:**
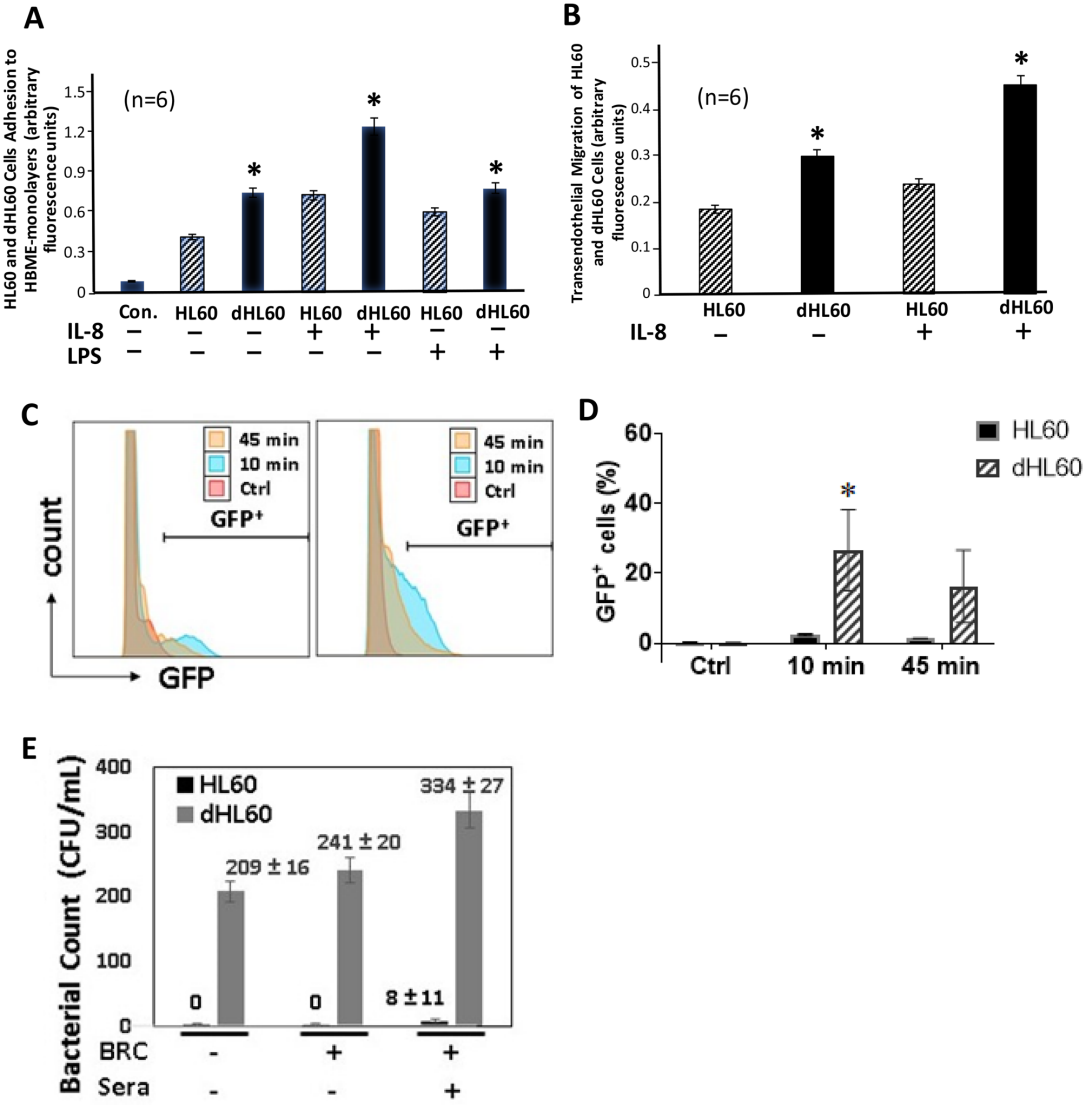
Cell Adhesion to and Migration across HBME Monolayers and Phagocytosis of GFP-Expressing *P. aeruginosa* (**A**) HMBEs were cultured to postconfluence in 24-well plates. The HBME monolayers were pretreated for 4 h with LPS 200 ng/ml or medium alone and gently washed. Equivalent concentrations of calcein AM-labeled HL60 and dHL60 cells, after pretreatment for 2 h with IL-8 3 nM or medium alone, were washed and co-incubated for 0.5 h with HBME monolayers. After gentle washing to remove nonadherent cells, the attached cells were fluorometrically assayed (excitation 485 nm, emission 530 nm) in a fluorescence plate reader. (**B**) HBMEs were cultured to postconfluence on the filter supports of transwell chambers. Equivalent concentrations of viable calcein AM-labeled HL60 and dHL60 cells were introduced into the upper compartment whereas IL-8 or medium alone was placed in the lower compartment and the transwell chambers incubated for 2 h at 37 °C. After incubation for 2 h, each compartment of each chamber was sampled and labeled cells fluorometrically assayed. Vertical bars represent mean ± SE adhesion/migration. (**C**, **D**). The nondifferentiated and DMF-differentiated HL60 cells were mixed with PAK-GFP at a MOI of 25, incubated for 10 or 45 min, and fixed for flow cytometric analysis. The cells alone served as negative control (Ctrl). The histograms (**C**) and the percentage of GFP-positive cells (**D**) are shown. (**E**) Non-opsonized (BRC^−^/sera^−^) and BBRC^−^ -and BRC^+^ sera^+^-opsonized *P. aeruginosa* were mixed with either nondifferentiated or DMF-differentiated HL60 cells, incubated at 37 °C for 45 min, and the mixtures sampled for CFUs. *, significantly increased adhesion, migration, or opsonophagocytosis in dHL60 cells compared with that seen for HL60 cells at *p* < 0.05. The data generated in each panel represents experiments performed on ≥ 2 independent occasions.

We previously established that PMN-endothelium interactions can be SA-dependent^10,11^. Since HL60 cell NEU activity and expression dramatically change during differentiation (Figs. 3, 5, and 6), we compared adhesion of HL60 and dHL60 cells to HBME monolayers (Fig. 10A). Adhesion of unstimulated dHL60 cells was 1.7-fold greater than was adhesion of unstimulated HL60 cells to unstimulated HBME monolayers. Similarly, adhesion of IL-8-stimulated dHL60 cells was 1.8-fold greater than adhesion of IL-8-stimulated HL60 cells to unstimulated HBMEs. Finally, adhesion of unstimulated dHL60 cells to LPS-activated HBMEs was > 1.3-fold greater compared to their adhesion to resting HBMEs. These combined data indicate that under all 3 experimental conditions, including resting HL60 cells with either resting or activated HBMEs, and activated HL60 cells with resting HBMEs, dHL60 cells were consistently more adherent to the endothelial surface than were HL60 cells.

### Migration of HL60 and dHL60 cells across post-confluent HBME monolayers

Since surface sialylation and maturation of myeloid cells each influence their ability to exit the bone marrow^1–3^, we studied the migration of HL60 and dHL60 cells across postconfluent HBME monolayers, in the presence or absence of an IL-8 chemotactic gradient (Fig. 10B). In the absence of an IL-8 gradient, transendothelial migration of dHL60 cells was > 1.6-fold greater than that seen for HL60 cells. The introduction of IL-8 increased migration of either HL60 or dHL60 cells compared to the same cells in the absence of IL-8 (Fig. 10B). In the presence of the IL-8 gradient, transendothelial migration of dHL60 cells was 1.9- fold greater than that seen by HL60 cells (Fig. 10B). These combined data indicate that the ability of dHL60 cells to adhere to and migrate across the HBME barrier exceeds that seen for HL60 cells. Whether these changes in HL60/dHL60 cellular migration can be explained indirectly through altered cell adhesion to the endothelium (Fig. 10A), an early prerequisite step for transendothelial migration, is unclear.

### Bacterial uptake in HL60 and dHL60 cells

GFP-expressing *P. aeruginosa* strain PAK was avidly associated with the dHL60 cells, but not the nondifferentiated HL60 cells (Fig. 10C, D). At 10 min, almost 25% of dHL60 cells contained the GFP compared to < 5% for nondifferentiated HL60 cells (Fig. 10D). The reduced GFP signal at 45 min likely represents degradation of the ingested bacteria. To confirm the lack of bacterial uptake by nondifferentiated HL60 cells by flow cytometric analysis, we added bacteria opsonized in baby rabbit complement (BRC) alone or combined with rabbit anti-PA 06 antisera, as well as non-opsonized bacteria to the nondifferentiated and dHL60 cells (Fig. 10E). The dHL60 cells ingested non-opsonized bacteria and this uptake increased with opsonization with BRC and increased still further in the presence of BRC and antisera. In contrast, the nondifferentiated HL60 cells were unable to take up bacteria under these same conditions. These results confirm the poor bacterial uptake by nondifferentiated HL60 cells observed by flow cytometric analysis (Fig. 10C, D).

## Discussion

Over 25 years ago, we established that mature human PMNs express NEU activity^31^. This catalytic activity could be localized to not only the primary and secondary granule subpopulations but also to the plasma membrane fraction. Several years later, human PMNs were reported to contain 2 distinct NEU activities: a freeze–thaw labile lysosomal activity for the 4-MU-NANA substrate, and a freeze–thaw stable activity for ganglioside substrates associated with the plasma membrane^54^. In the current studies in unstimulated resting PMNs, we found relatively modest levels of total NEU activity compared to those NEU activities reported in human airway epithelia^42,45^, human lung microvascular endothelia^45,46^, and human lung fibroblasts^45,47^. However, it is conceivable that relatively high levels of one or more NEUs could be strategically localized to one or more PMN subcellular compartment(s), such as the leading edge of a migrating cell, where it is most needed. In fact, activated PMNs co-cultured with endothelial monolayers have been shown to desialylate the endothelial surface^10^. In PMNs, we could only detect NEU2 but not NEU1, PPCA, NEU3, or NEU4 proteins. Although PMNs were assayed for NEU activity in the presence of protease inhibitors, and directly lysed in SDS-containing sample buffer for immunoblotting studies, that protease-rich PMNs manipulated in vitro for their isolation, might proteolytically degrade NEU enzymes and reduce their NEU activity, cannot be absolutely excluded.

To gain insight into NEU biology and expression in mature PMNs, we studied NEU activity and expression in the HL60 cell line, and changes that might occur during their differentiation into PMN-like cells. We found that total NEU activity decreased in differentiating HL60 cells. We asked whether the changes in NEU activity during HL60 cellular differentiation could be explained through altered NEU and/or PPCA expression. Since no consistent changes in NEU1, PPCA, or NEU3 mRNA expression were evident and NEU2 and NEU4 mRNAs appeared to modestly increase only on day 7, we assessed their protein expression over the same 7-day time window. NEU1 protein profoundly decreased, whereas PPCA protein expression dramatically increased. That PPCA protein expression continues to increase while NEU1 protein expression becomes almost undetectable, raises the possibility that PPCA functions as more than solely a NEU1 chaperone. We have established discordant NEU1 and PPCA expression in other cell types, including human small airway epithelia, lung microvascular endothelia, and lung fibroblasts^45^. In addition to serine carboxypeptidase activity, PPCA also displays deamidase and esterase activities^55^. PPCA exerts protective activity not only for NEU1, but for β-galactosidase as well^55^. Its catalytic and protective activities are distinct from one another. Whether one or more of these reported PPCA activities, and/or an as yet unknown PPCA function might be operative during HL60 differentiation, is unknown. NEU2 and NEU4 protein expression were also elevated in dHL60 cells compared to HL60 cells. NEU3 remained at the same level throughout. Perhaps the differentiation-driven decrease in NEU activity for the 4MU-NANA substrate can be explained, in part, through down-regulation of NEU1 expression. It is conceivable that the increases in NEU2 and NEU4 protein were not sufficient to compensate for the decrease in NEU1 and/or were less catalytically active for the 4-MU-NANA substrate.

In the current studies with proapoptotic PMNs and differentiating HL60 cells, we have demonstrated upregulation of NEU2 protein expression. NEU2 gene expression has been reported in unspecified leukocytes^56^ and in IL-1β-stimulated PMNs^57^. During murine myoblast differentiation, NEU2 expression was upregulated, and forced overexpression of rat NEU2 in these same cells, decreased cell proliferation and promoted spontaneous myoblast differentiation with formation of myogenin-positive myotubes^58^. In the chronic myelogenous leukemia K562 cell line, NEU2 expression in these rapidly proliferating cells was suppressed^59^. Forced NEU2 overexpression in these same cells reduced their proliferation rate and increased their susceptibility to apoptosis. In the current studies with mature PMNs and differentiating HL60 cells, increasing NEU2 expression was temporally coincident with these same features, decreased cell numbers and loss of viability. Pretreatment of proapoptotic PMNs with G-CSF down-regulated their NEU2 protein expression, and at the same time, protected against their undergoing apoptosis. It is conceivable that this elevated NEU2 protein expression in either PMNs or dHL60 cells explains, in part, their growth arrest and loss of viability. Upon departure from the bone marrow, mature postmitotic PMNs display an extremely brief intravascular half-life^4,30^. Their elevated NEU2 protein expression might dictate their accelerated apoptosis^4,30^.

The surface sialylation of mammalian cells, including PMNs, can mask plasma membrane changes of apoptosis, such as externalization and exposure of phosphatidylserine, as well as other surface structures, such as calreticulin, both of which serve as “eat me” signals to monocyte-derived phagocytes^60,61^. Cells undergoing apoptosis and membrane blebbing lose their surface-expressed α2,3- and α2,6-linked sialic acid residues, permitting their engulfment by these same phagocytic cells^60^. Treatment of cells with exogenous neuraminidase to remove sialic acid, or masking these same sugar residues with sialic acid-binding lectins, SNA and MALI, each enhanced their engulfment^60^. In the current studies, PMNs cultured in vitro displayed a profound elevation of NEU2 protein expression in parallel with proapoptotic changes. We asked whether this elevated NEU2 expression might be associated with PMN surface desialylation, a hallmark of apoptosis^60,61^. The increased NEU2 expression was temporally coincident with PMN surface desialylation. These findings raise the question as to whether NEU2-mediated plasma membrane desialylation may be a key component of the apoptotic process. However, due to technological limits in manipulating NEU2 expression in PMNs, a causal link between NEU2 catalytic activity and PMN surface desialylation and/or apoptosis could not be definitively established.

With dramatic changes in NEU1, PPCA, NEU2, and NEU4 protein expression during HL60 differentiation, we asked whether altered sialylation over the same time period might ensue. MAL lectin blotting of resolved dHL60 cell lysates revealed MAL-reactive bands that, over the 7-day differentiation period, lost their intensity compared to that seen with HL60 cells. It is conceivable that these differentiation-provoked decreases in α2, 3-linked sialylation could be explained through NEU2-mediated desialylation. In fact, NEU2 reportedly hydrolyzes bonds for α2,3-linked SA at a Vmax ~ sevenfold higher than that seen for α2,6-linked SA^62–64^. This substrate preference that NEU2 exerts might explain, in part, the decrease in α2,3-linked sialylation without a comparable decrease in α2,6-linked sialylation. However, the decrease in α2,3-linked sialylation was not prevented by broadspectrum NEU inhibition with 2DN. MALDI ToF MS spectral analysis of HL60 and dHL60 cells revealed that, indeed, α2,6-linked SA substitutions were selectively increased in dHL60 cells. In support of our findings of decreased α2, 3-linked sialylation, Gadhoun et al. reported that introduction of exogenous NEUs known to exhibit preference for α2, 3-linked sialylation to HL60 cell cultures, reproduced the sialylation changes observed with differentiation^5^. Over the same 7-day differentiation period, while α2,3-linked sialylation decreased, SNA lectin blotting revealed increased α2,6-linked sialylation in dHL60 cells compared to HL60 cells. Similarly, Le Marer et al. used SNA lectin flow cytometry to show increased surface α2,6-linked sialylation on maturing human bone marrow-derived myeloid cells^7^. The profound decrease in NEU1 expression could contribute to this increased sialylation. Since at least some of the SNA-reactive bands were also PNA-reactive, these combined data are compatible with transfer of SA to galactose in α2, 6-linkage.

Only two STs are known to transfer SA to galactose in α2, 6-linkage, ST6GAL-1 and ST6GAL-2^24,25^. Using qRT-PCR, ST6GAL-1 mRNA was detected in HL60 cells, but with differentiation, dramatically decreased. Taniguchi et al. also found that HL60 differentiation in response to either DMSO or RA down-regulated ST6GAL-1 mRNA levels^6^. ST6GAL-2 mRNA was also detected but at levels < 7% of that seen for ST6GAL-1. Several studies have failed to detect ST6GAL-2 mRNA in peripheral blood leukocytes using Northern analysis^65,66^. In contrast to ST6GAL-1, ST6GAL-2 mRNA levels increased with differentiation. Although ST6GAL-1 displays a ubiquitous tissue expression pattern^67,68^, ST6GAL-2 mRNA expression is reportedly restricted to the brain^66^. Accordingly, we had not anticipated ST6GAL-2 mRNA expression in HL60 cells. That neither ST6GAL-1 nor ST6GAL-2 protein increased and CMP failed to block the increased α2, 6-linked sialylation, taken together, do not support an ST-mediated process.

We^10,11^ and others^7,8^ have previously reported that changes in myeloid cell sialylation influence their adhesion to and migration across the endothelium. We previously found that prior treatment with exogenous NEU of either resting human PMNs or bovine/human pulmonary vascular endothelia, increased PMN adhesion to and migration across post-confluent endothelial monolayers^10^. In these same studies, prior NEU inhibition with 2DN diminished fMLP-activated PMN adhesion to and migration across TNFα-stimulated endothelia. In another study, we found that neutralizing anti-NEU antibodies dramatically reduced pulmonary leukostasis in mice in response to systematic complement activation, or PMN recruitment to the bronchoalveolar compartment in response to intratracheal installation of IL-8^13^. In still yet another study, we found that prior intratracheal installation of exogenous NEU enhanced LPS-induced PMN recruitment into BALF^37^. Since HL60 differentiation includes changes in NEU protein expression, NEU activity, and both α2,3-linked and α2,6-linked sialylation, we asked whether these changes generated functional consequences. We found that dHL60 cells displayed greater adhesion to and migration across post-confluent HBME monolayers than did nondifferentiated HL60 cells. That dHL60 cells contain profoundly reduced NEU activity, which in turn allows for increased surface sialylation, but at the same time exhibits greater adhesion, may implicate sLex- selectin interaction(s). However, in our static experimental system, selectins are less likely to be operative. Finally, dHL60 cells exhibited greater bacterial uptake/phagocytosis than did HL60 cells. Taken together, HL60 cells differentiated towards PMNs display greater adhesion to and migration across the endothelial barrier, and possibly after entering extravascular tissues, enhanced phagocytic potential.

The HL60 leukemic cell line differentiated towards PMN-like cells has been used as a surrogate for mature human PMNs^5–8^. In the context of NEU expression, dHL60 cells and mature PMNs express comparable levels of NEU activity for the 4-MU-NANA substrate. The two cell types both express high levels of PPCA and NEU1 mRNAs and low, almost undetectable levels of NEU2, NEU3, and NEU4 mRNAs. At the protein level, dHL60 cells express high levels of NEU2 and PPCA, and lower levels of NEU1, NEU3, and NEU4. In contrast, mature PMNs only express NEU2 but not NEU1, PPCA, NEU3 or NEU4 proteins. Such differences in NEU expression raise the question as to whether HL60 cells differentiated towards PMN-like cells can consistently provide a faithful surrogate for mature PMNs. Since HL60 cell function dramatically changes with differentiation, it is critical that when these cells are utilized in functional assays, the differentiating agent and duration of differentiation be carefully controlled and standardized.

A number of studies have provided mechanistic explanations for the changes in myeloid cell- endothelial cell interactions associated with differentiation^5,7,8^. During HL60 differentiation, β1 integrin expression increases and its desialylation enhances cell adhesion to fibronectin^8^. In another study, HL60 differentiation increases NEU activity for α2,3-linked sialylation, which in turn, converts sLe^x^/CD15s to Le^x^/CD15^5^. In still another study, during maturation of bone marrow-derived myeloid cells, increased α2,6-linked sialylation of CD11b and CD18 reduced cell adhesion to fibronectin and bone marrow stroma^7^. We previously reported that endogenous PMN NEU activity desialylates surface-expressed CD11b and CD18, which in turn, increases PMN binding to intercellular adhesion molecule (ICAM)-1 and -2 on the endothelial surface^11^. Desialylation of several PMN surface-expressed glycoproteins, including CD43, CD44, and CD16, increases their susceptibility to matrix metalloprotease-mediated proteolysis, and alters their adhesive function^17,69^. It is conceivable that the changes in NEU protein expression and sialylation in mature, differentiated myeloid cells might explain their superior adhesive and migratory functions, and their increased ability to egress the bone marrow sinusoids. Changes in PMN surface sialylation, in turn, can regulate accessibility of both inhibitory SA binding immunoglobulin-like lectin (siglec)s, such as siglecs 5 and 9^70^, and β-galactoside-binding lectins, i.e. galectins, such as galectins 1 and 3^71^ to the PMN surface. Engagement of these siglecs and galectins with the PMN surface is known to profoundly influence PMN behavior, including myeloid differentiation^72^. Finally, the identification of which specific NEU(s) participates in specific signaling pathways and cellular processes should provide targets for therapeutic intervention with recently described^45,73^ selective NEU inhibitors.

## Supplementary Information

Supplementary Information.
