# Supplementary material for: Altered sialidase expression in human myeloid cells undergoing apoptosis and differentiation
Source: Sci Rep. 2022 Aug 19;12:14173. doi: 10.1038/s41598-022-18448-6 (PMC9390117; doi:10.1038/s41598-022-18448-6)
Supplement: Supplementary file 1 — Supplementary Information. [file 41598_2022_18448_MOESM1_ESM.pdf]

Altered sialidase expression in human myeloid cells undergoing apoptosis and differentiation

Sang W. Hyun

Chiguang Feng

Anguo Liu

Erik P. Lillehoj

Rossana Trotta

Tami J. Kingsbury

Antonino Passaniti

Katerina N. Lugkey

Sitara Chauhan

John F. Cipollo

Irina G. Luzina

Sergei P. Atamas

Alan S. Cross

Simeon E. Goldblum

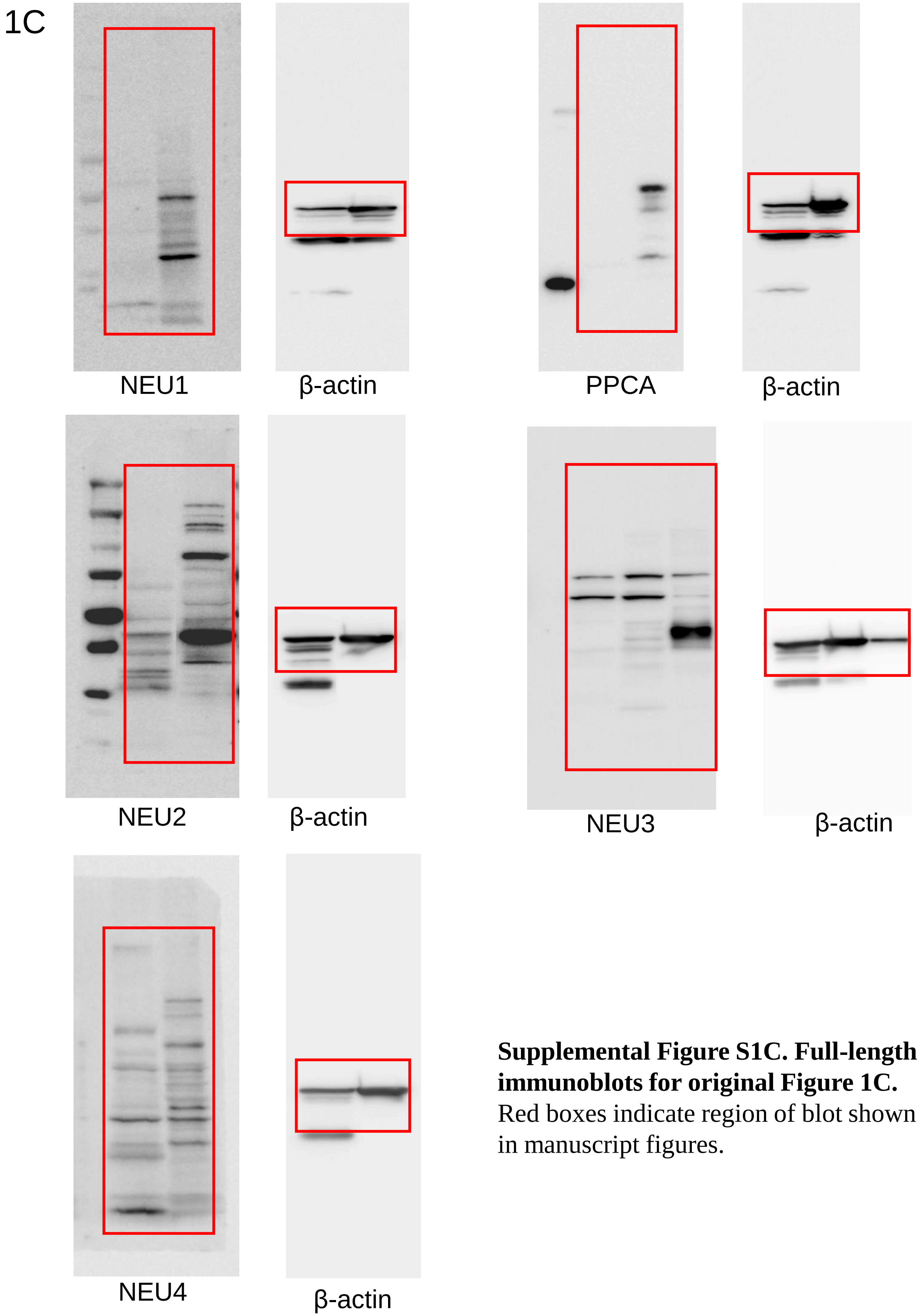

Supplemental Figure S1C. Full-length immunoblots for original Figure 1C. Red boxes indicate region of blot shown in manuscript figures.

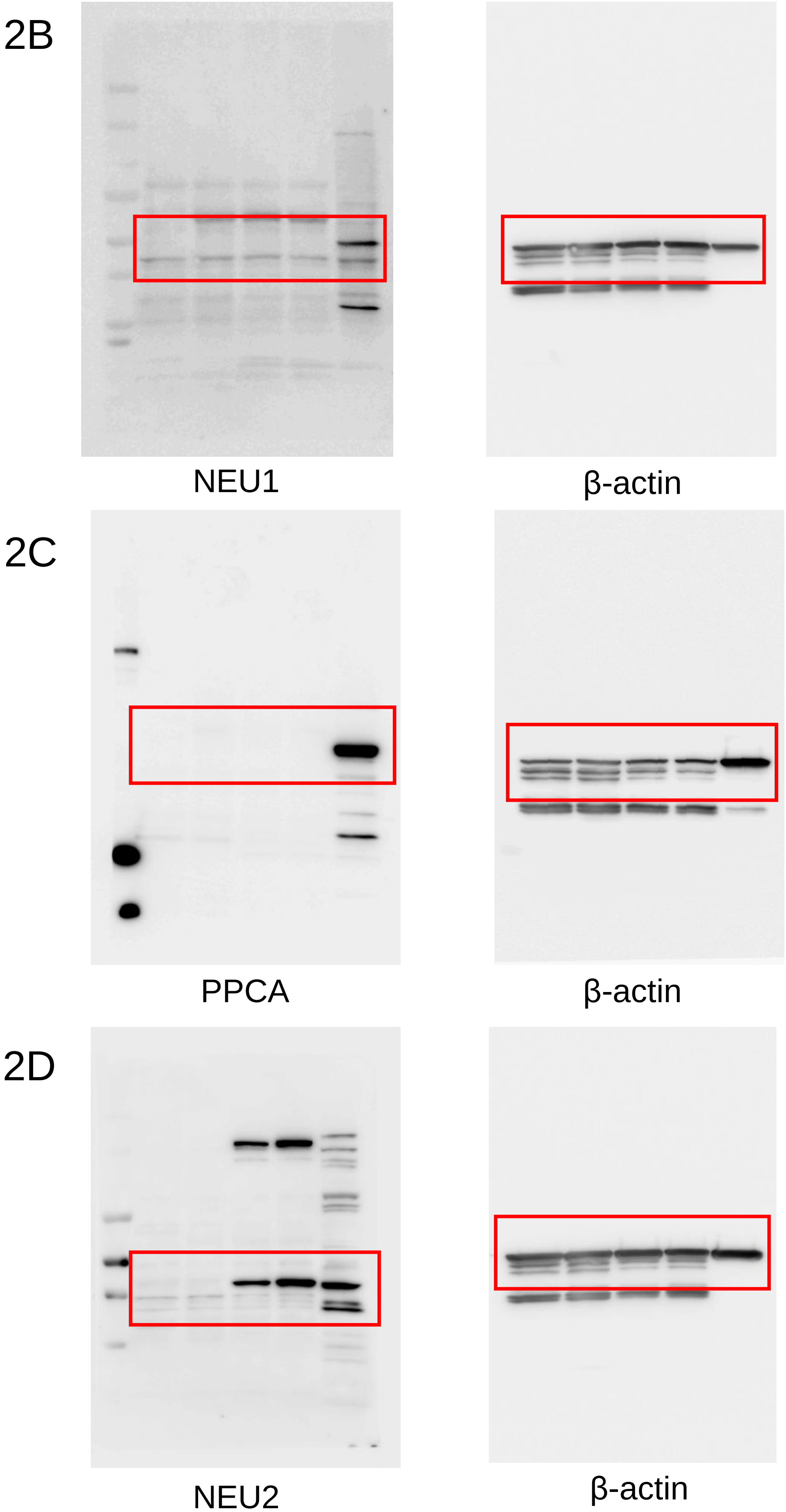

Supplemental Figure S2. Full-length immunoblots for original Figures 2B, 2C, and 2D. Red boxes indicate region of blot shown in manuscript figures.

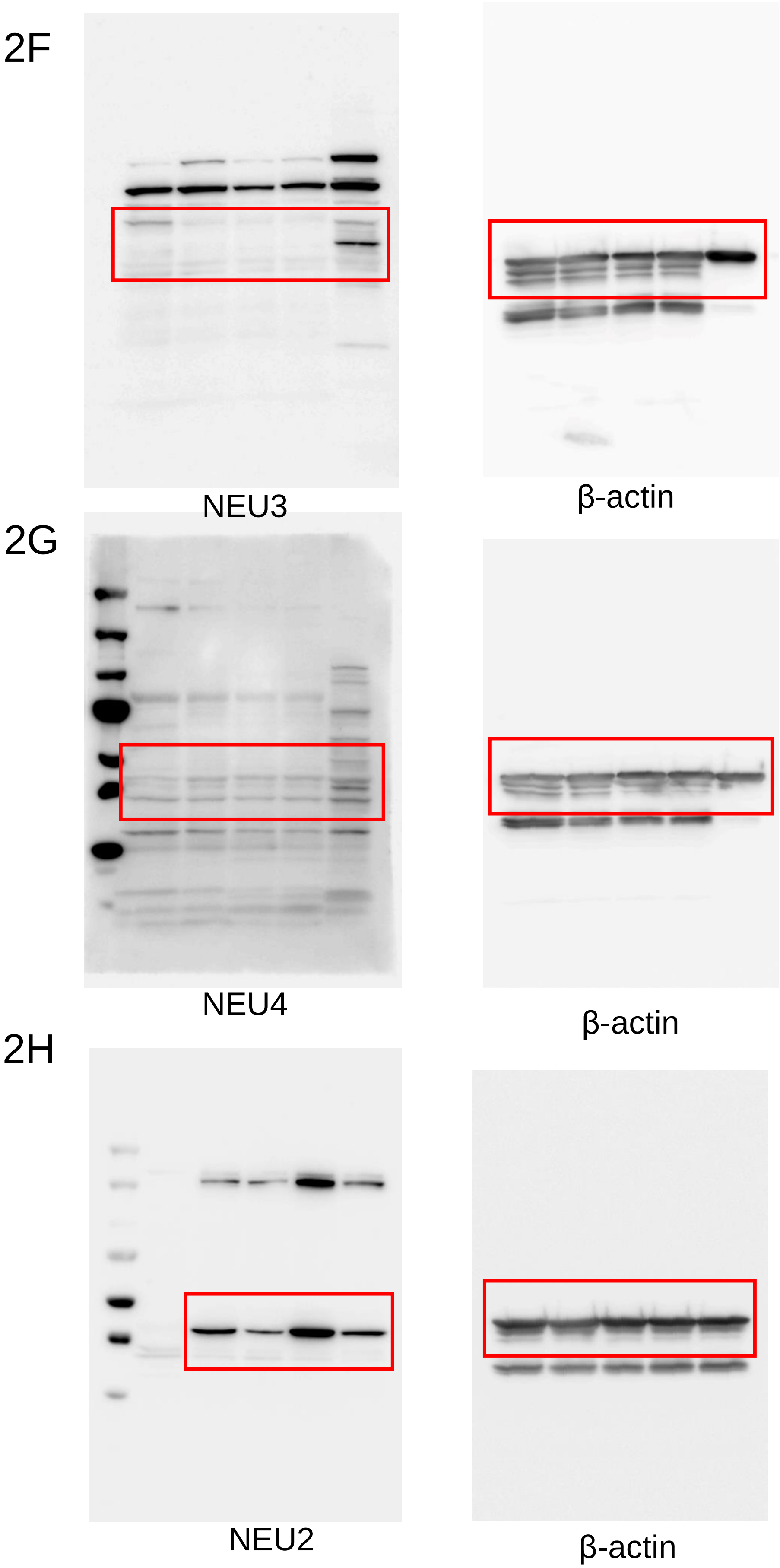

Supplemental Figure S2 (continued). Full-length immunoblots for original Figures 2F, 2G, and 2H. Red boxes indicate region of blot shown in manuscript figures.

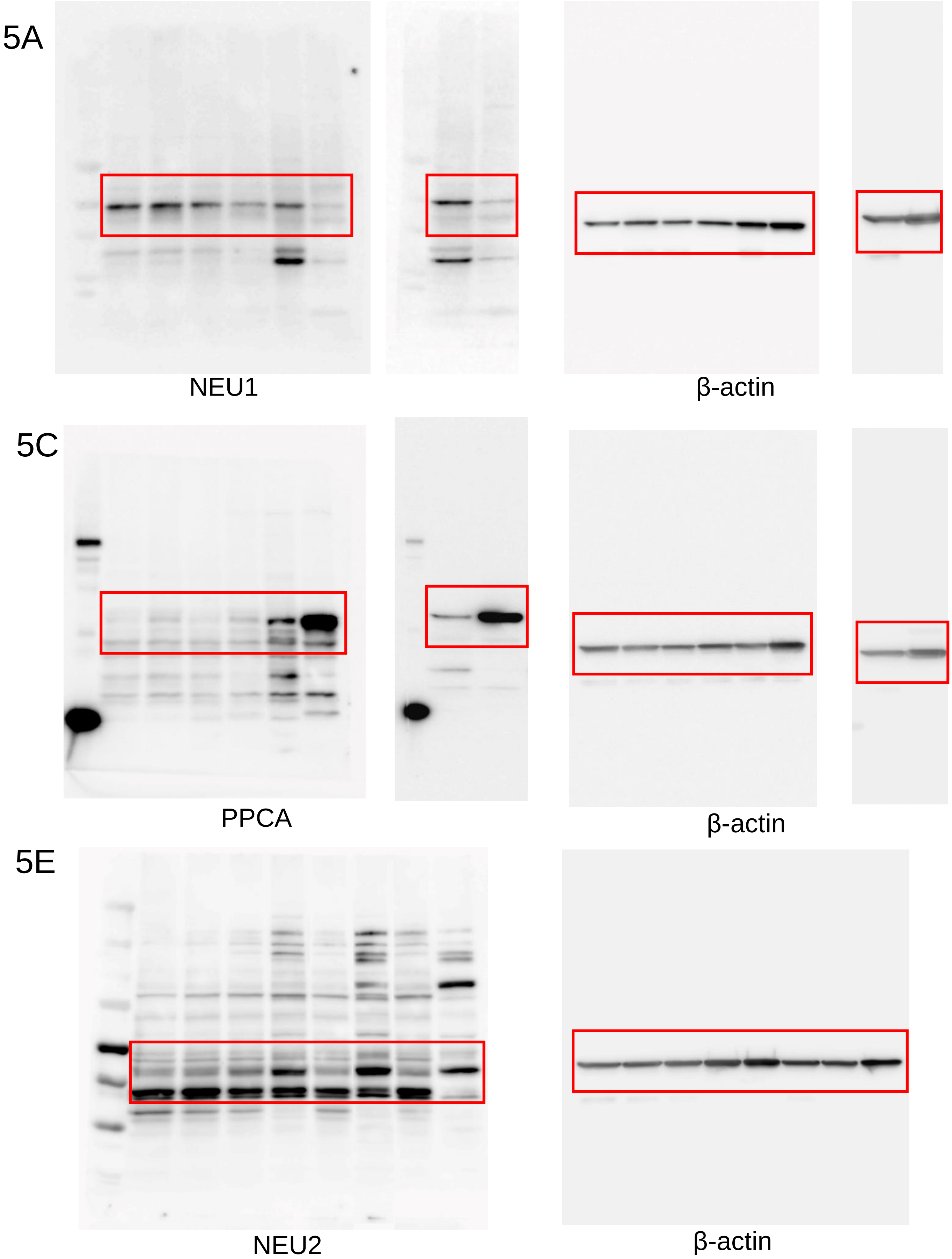

Supplemental Figure S5. Full-length immunoblots for original Figures 5A, 5C, and 5E. Red boxes indicate region of blot shown in manuscript figures.

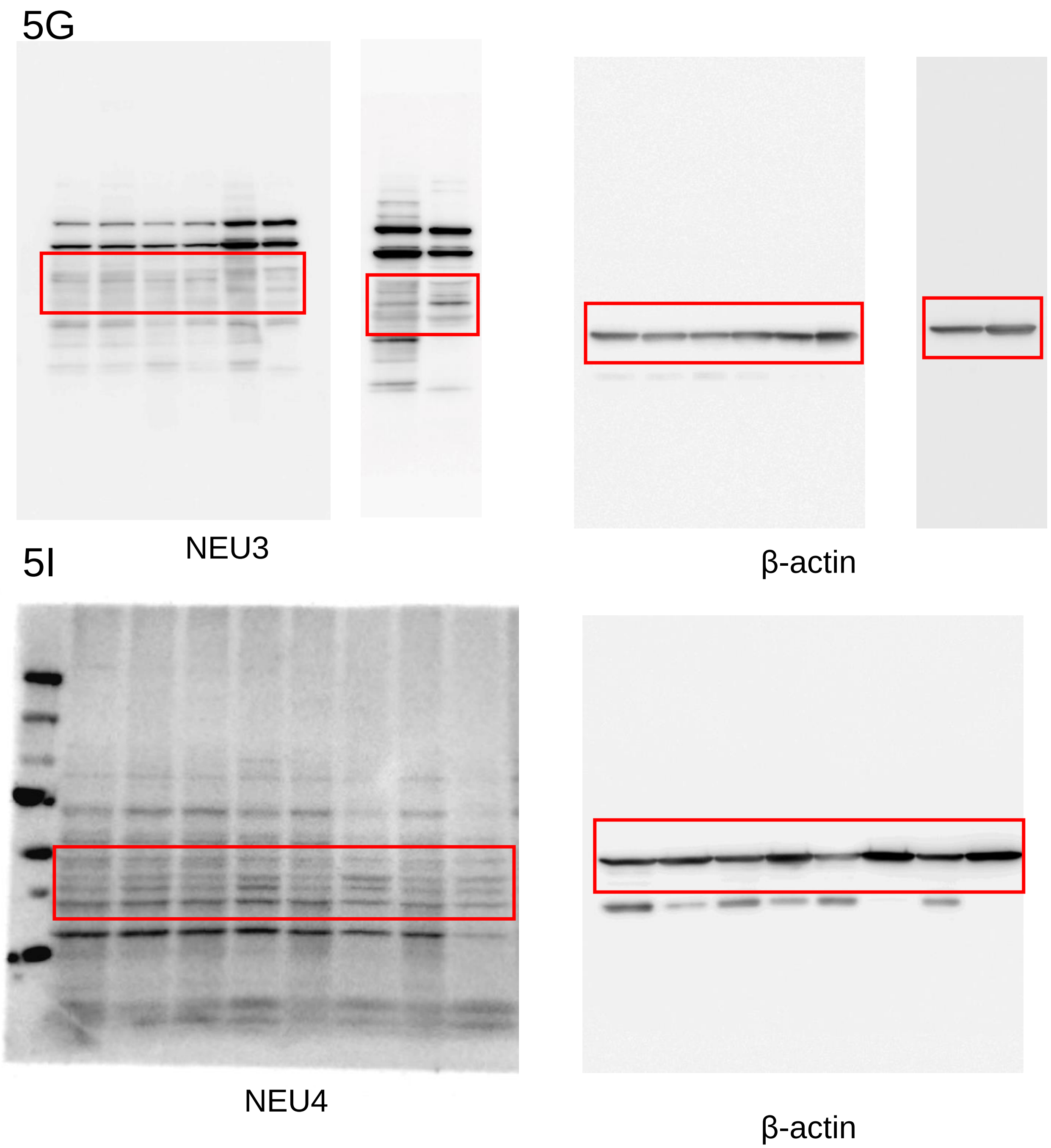

Supplemental Figure S5 (continued). Full-length immunoblots for original Figures 5G and 5I. Red boxes indicate region of blot shown in manuscript figures.

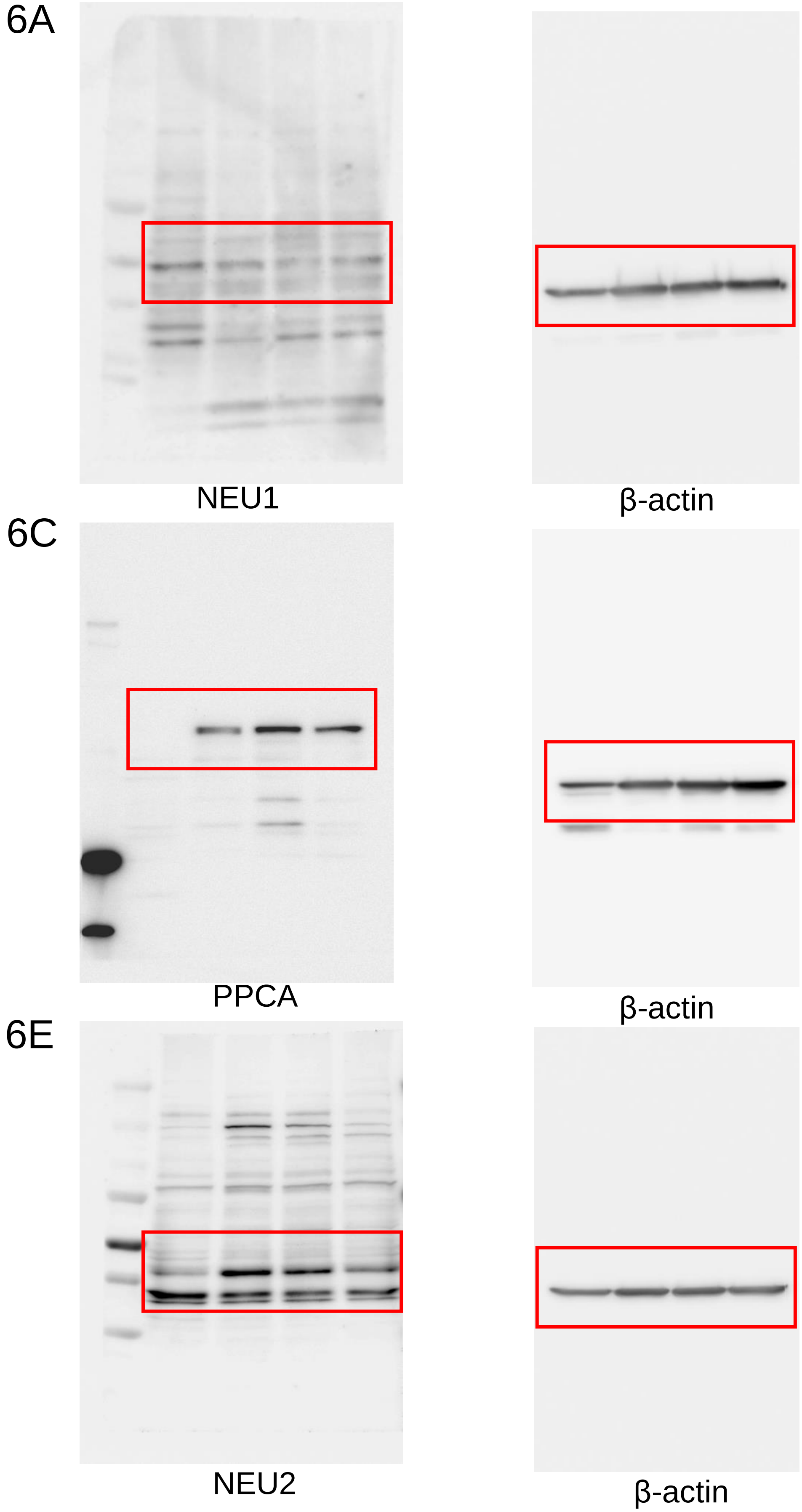

Supplemental Figure S6. Full-length immunoblots for original Figures 6A, 6C, and 6E. Red boxes indicate region of blot shown in manuscript figures.

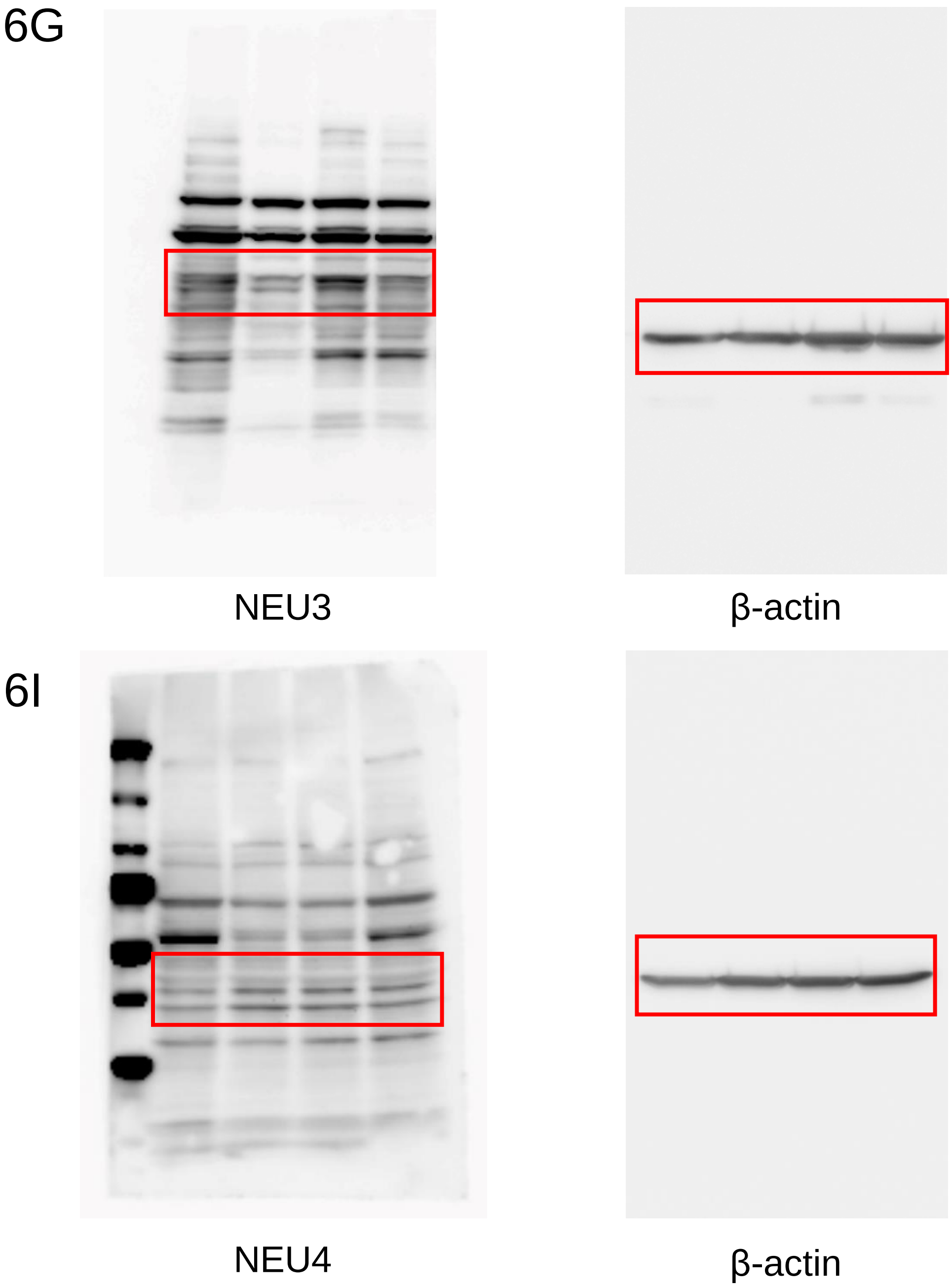

Supplemental Figure S6 (continued). Full-length immunoblots for original Figures 6G and 6I. Red boxes indicate region of blot shown in manuscript figures.

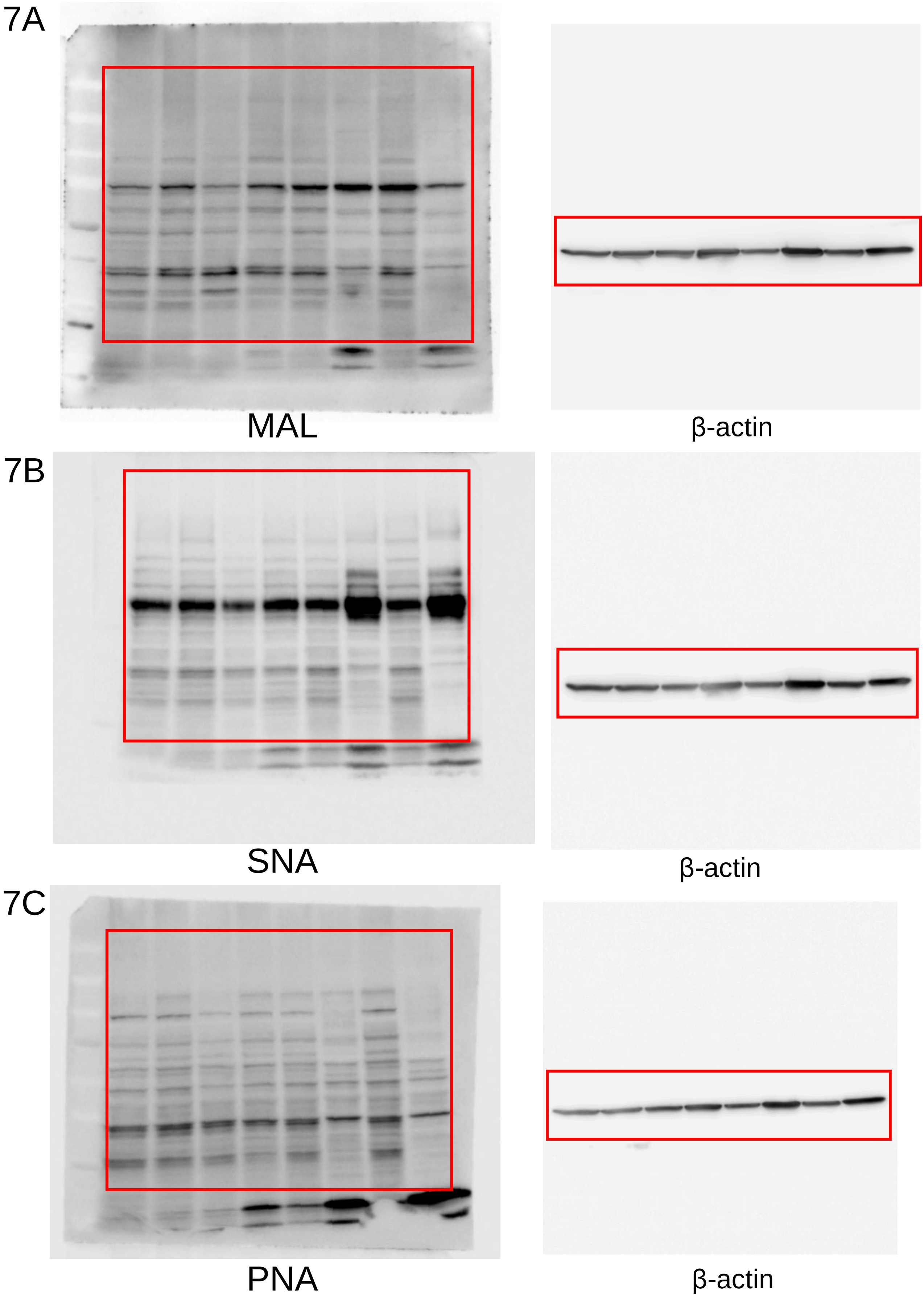

Supplementary Figure S7. Full-length immunoblots for original Figures 7A, 7B, and 7C. Red boxes indicate region of blot shown in manuscript figures.

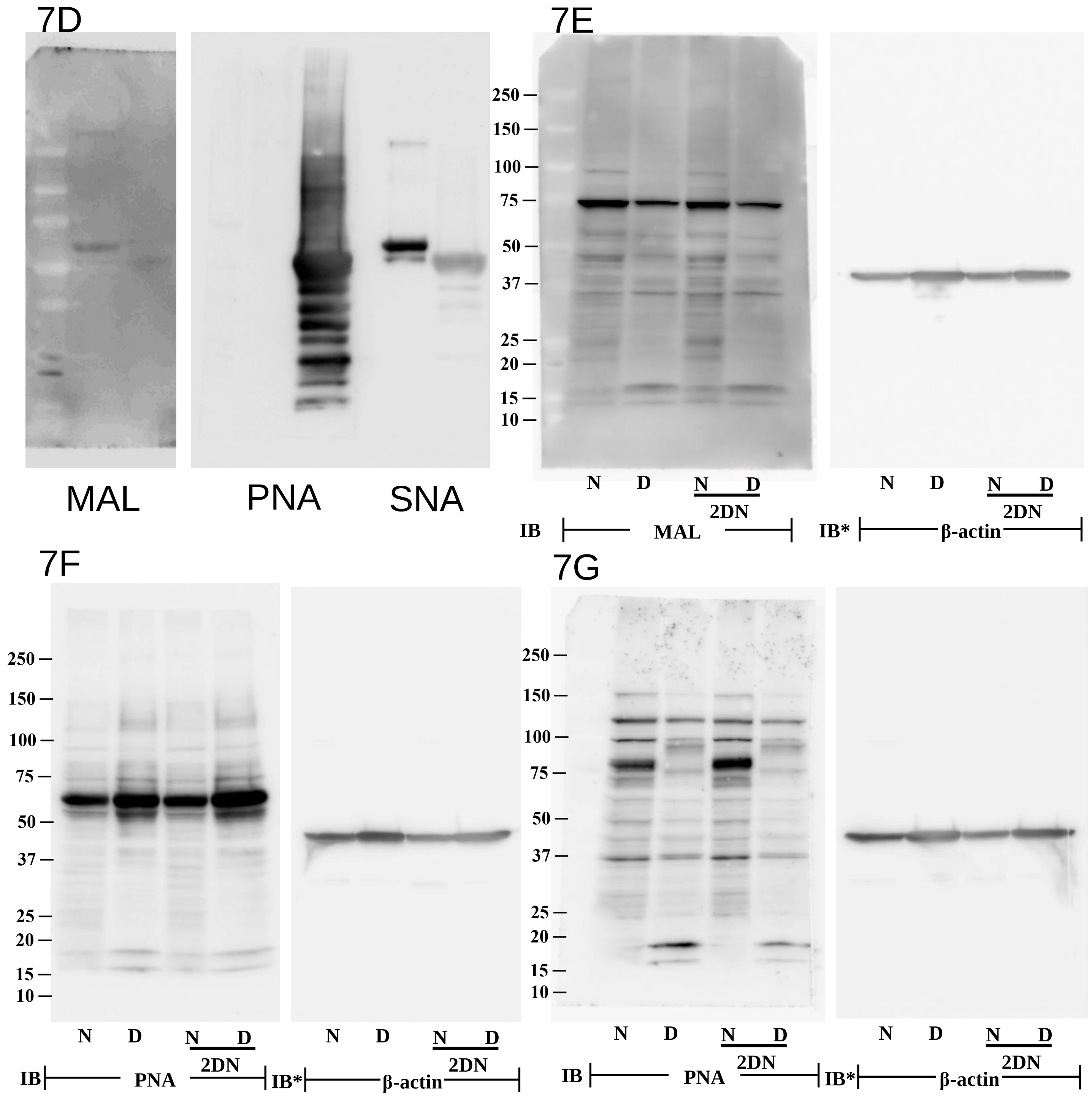

Supplementary Figure S7 (continued). Changes in HL60 cell sialylation during differentiation. (D) Fetuin and asialofetuin (1.0 µg) were processed for MAL, SNA, and PNA lectin blotting to validate lectin selectivity. (E-G) HL60 cells were cultured for 7 days with DMF or medium alone in the presence or absence of 2DN, lysed, and the lysates processed for lectin blotting with biotinylated MAL (E), SNA (F), or PNA (G). To control for protein loading and transfer, blots were stripped and re-probed for β-actin. MW in kDa indicated on the left. N = nondifferentiated, D = differentiated, 2DN = 2-deoxy-NANA. Each lectin blot is representative of 2 independent experiments.

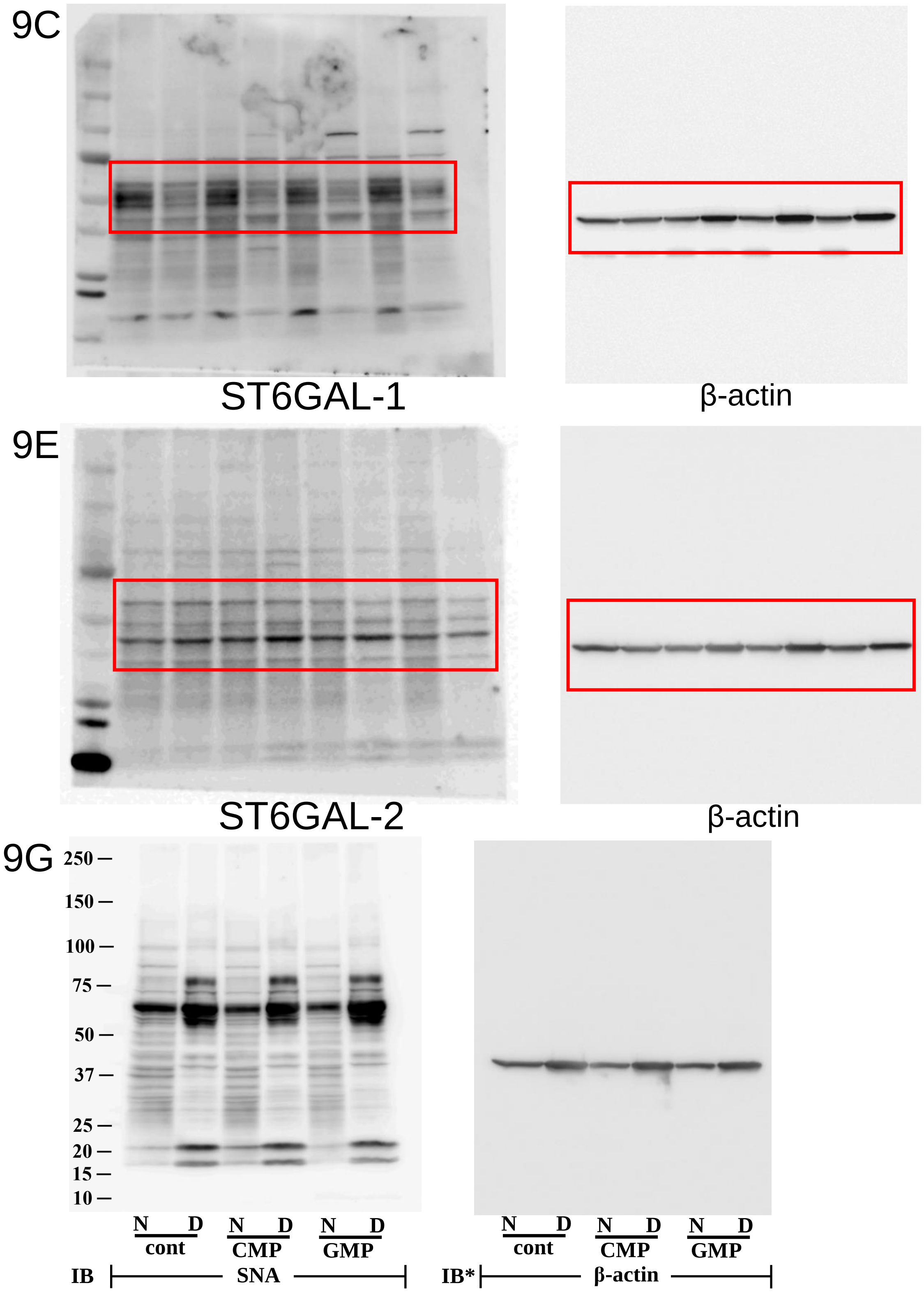

Supplemental Figure S9. Broad-spectrum ST inhibition with CMP fails to prevent HL60 cell differentiation-induced increases in α2,6-linked sialylation. (C, E) Full-length immunoblots for original Figures 9C and 9E. (G) HL60 cells were cultured in the presence of DMF (D) or medium alone (N). In addition, CMP, GMP, or medium alone were introduced. At 7 days, the cells were lysed and the lysates processed for SNA lectin blotting. To control for protein loading and transfer, blots were stripped and re-probed for β- actin. IB = immunoblot, IB* = immunoblot after stripping. MW in kDa is indicated on the left.

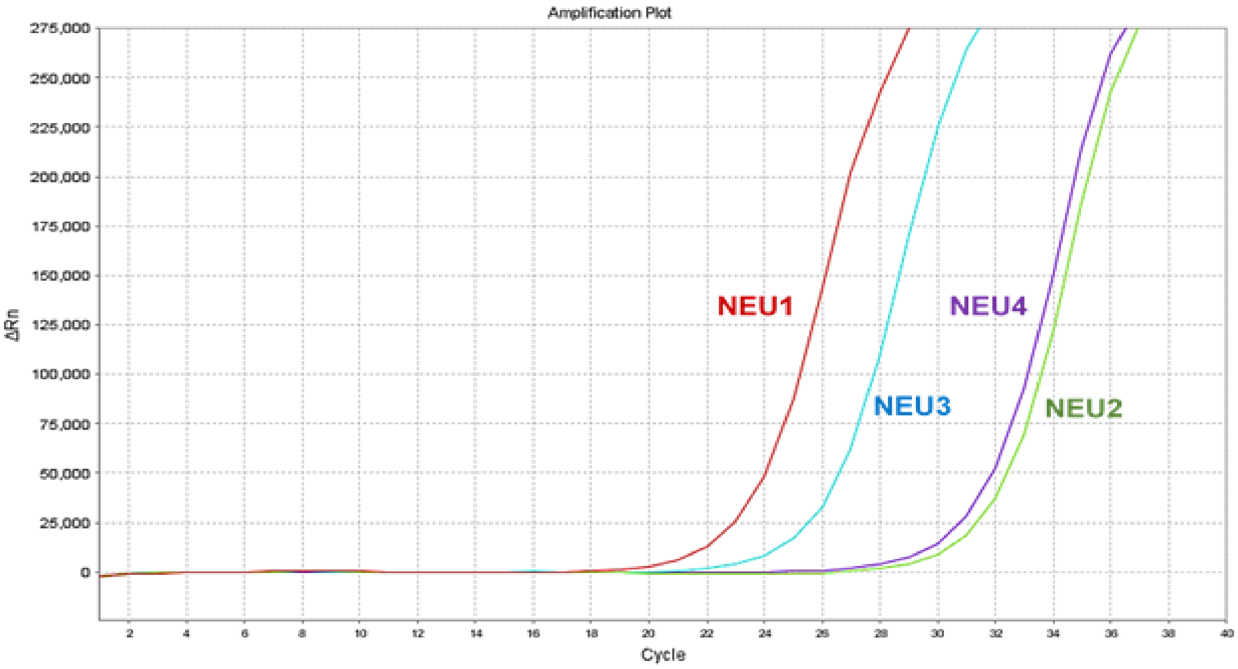

Supplemental Figure S11. Amplification curves with primers for NEU1-4 in the same sample, revealing parallel curves indicative of similar amplification rates for these 4 targets.

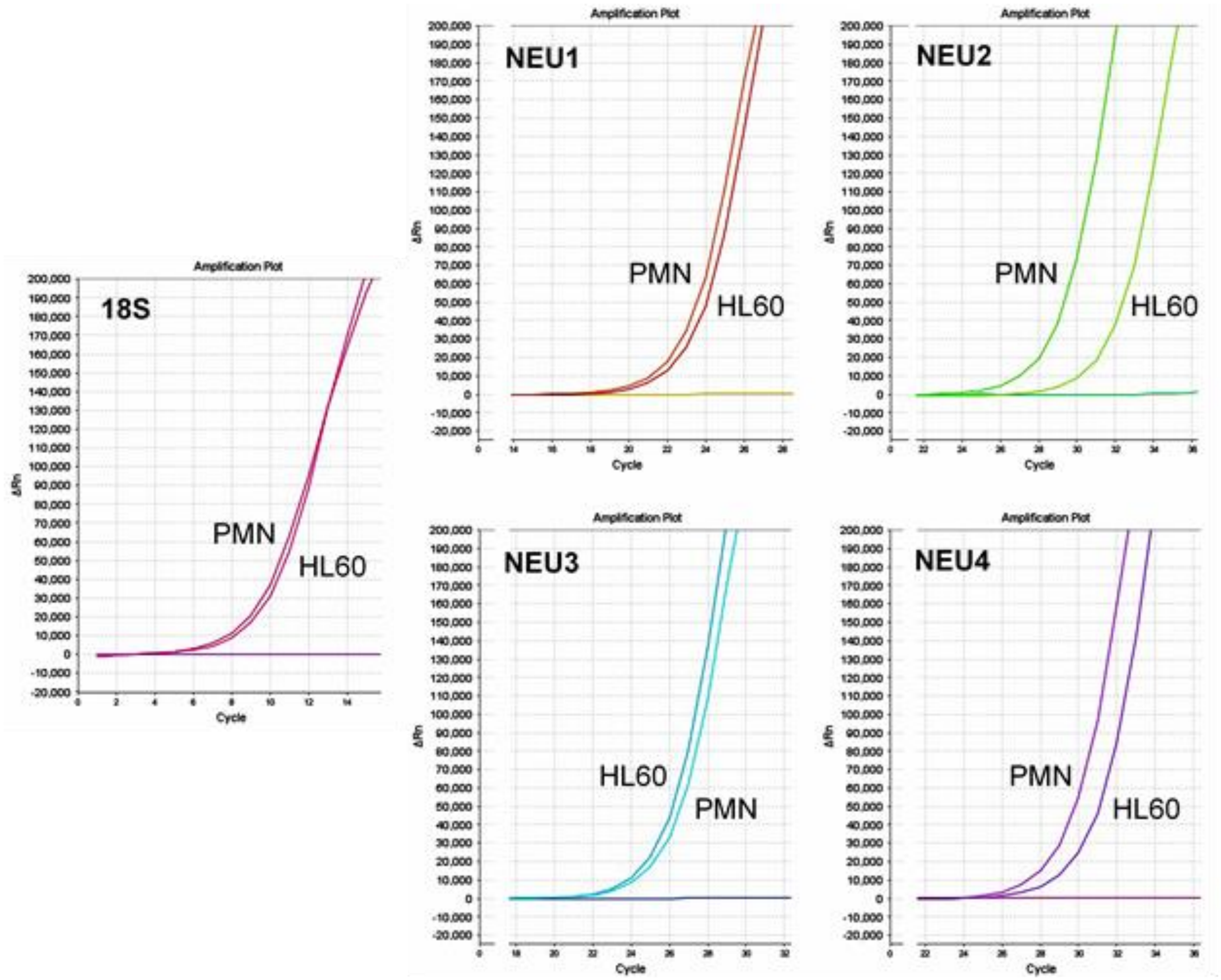

Supplemental Figure S12. Amplification curves with primers for 18S and NEU1-4 in PMNs and HL60 cells analyzed in the same experiments.
